# Supplementary material for: Small heat shock proteins operate as molecular chaperones in the mitochondrial intermembrane space
Source: Nat Cell Biol. 2023 Jan 23;25(3):467–80. doi: 10.1038/s41556-022-01074-9 (PMC10014586; doi:10.1038/s41556-022-01074-9)
Supplement: Supplementary file 1 — Reporting Summary [file 41556_2022_1074_MOESM1_ESM.pdf]

nature portfolio

Corresponding author(s): Vincent Timmerman

Last updated by author(s): Sep 9, 2022

Reporting Summary

Nature Portfolio wishes to improve the reproducibility of the work that we publish. This form provides structure for consistency and transparency in reporting. For further information on Nature Portfolio policies, see our Editorial Policies and the Editorial Policy Checklist.

Statistics

For all statistical analyses, confirm that the following items are present in the figure legend, table legend, main text, or Methods section.

n/a	Confirmed	
☐	☒	The exact sample size (*n*) for each experimental group/condition, given as a discrete number and unit of measurement
☐	☒	A statement on whether measurements were taken from distinct samples or whether the same sample was measured repeatedly
☐	☒	The statistical test(s) used AND whether they are one- or two-sided<br>*Only common tests should be described solely by name; describe more complex techniques in the Methods section.*
☒	☐	A description of all covariates tested
☐	☒	A description of any assumptions or corrections, such as tests of normality and adjustment for multiple comparisons
☐	☒	A full description of the statistical parameters including central tendency (e.g. means) or other basic estimates (e.g. regression coefficient) AND variation (e.g. standard deviation) or associated estimates of uncertainty (e.g. confidence intervals)
☐	☒	For null hypothesis testing, the test statistic (e.g. *F*, *t*, *r*) with confidence intervals, effect sizes, degrees of freedom and *P* value noted<br>*Give P values as exact values whenever suitable.*
☒	☐	For Bayesian analysis, information on the choice of priors and Markov chain Monte Carlo settings
☒	☐	For hierarchical and complex designs, identification of the appropriate level for tests and full reporting of outcomes
☒	☐	Estimates of effect sizes (e.g. Cohen's *d*, Pearson's *r*), indicating how they were calculated

Our web collection on statistics for biologists contains articles on many of the points above.

Software and code

Policy information about availability of computer code

Data collection	Confocal microscopy images were collected using ZEN software version 2022 Black SP7 FP3 (Carl Zeiss Microscopy, GmbH, Germany) connected to a LSM700 with release version 14.0.26.201.
Data analysis	1. FlowJo10 software (Tree Star Inc., Ashland, OR, USA) for FACS-data analysis.<br>2. MaxQuant (version 1.5.3.30 and version 2.1.0.0) for mass spectrometry data analysis.<br>3. PRISM 9 software (GraphPad Software, La Jolla, CA, USA) for statistical analysis and generate graphs.<br>4. ImageJ software (Schindelin et al. 2015) for immunofluorescence and electron microscopy image analysis.<br>5. Wave software version 2.6 (Agilent Technologies) for seahorse measurements.

For manuscripts utilizing custom algorithms or software that are central to the research but not yet described in published literature, software must be made available to editors and reviewers. We strongly encourage code deposition in a community repository (e.g. GitHub). See the Nature Portfolio guidelines for submitting code & software for further information.

Data

Policy information about availability of data

All manuscripts must include a data availability statement. This statement should provide the following information, where applicable:

- Accession codes, unique identifiers, or web links for publicly available datasets
- A description of any restrictions on data availability
- For clinical datasets or third party data, please ensure that the statement adheres to our policy

The Uniprot/Swiss-Prot database (database release version of April 2015) was accessed for proteomics data analysis. Proteomics from HSPB1-S135F affinity-enriched mass spectrometry has been deposited to the ProteomeXchange Consortium via the PRIDE partner repository with the dataset identifier PXD038275.

Source data are provided in Source data. All other data supporting the findings of this study are available from the corresponding author on reasonable request. Further information on the research design is available in the Nature Research Reporting Summary linked to this article.

Field-specific reporting

Please select the one below that is the best fit for your research. If you are not sure, read the appropriate sections before making your selection.

☒ Life sciences ☐ Behavioural & social sciences ☐ Ecological, evolutionary & environmental sciences

For a reference copy of the document with all sections, see nature.com/documents/nr-reporting-summary-flat.pdf

Life sciences study design

All studies must disclose on these points even when the disclosure is negative.

Sample size	No statistical methods were applied to pre-evaluate sample size. Experiments were performed at least as three replicates, according to current practices in the field. Statistical analysis was only performed on experiments for which the sample size included at least 3 biological replicates. Sample sizes were based on our previous experience (for microscopy see doi: 10.15252/embj.2019103811) and current standards in the field.
Data exclusions	No data were excluded from the analyses.
Replication	All experiments were replicated at least three times with similar findings. Sample sizes are provided in each figure legend.
Randomization	Samples were allocated into experimental groups by genotype and shRNA condition.Covariates were controlled for by maintaining all samples in the same growth and media conditions.
Blinding	The investigators were not blinded to treatment or genotype allocations in this study. For cell based experiments it was not possible to blind the experimenter.

Reporting for specific materials, systems and methods

We require information from authors about some types of materials, experimental systems and methods used in many studies. Here, indicate whether each material, system or method listed is relevant to your study. If you are not sure if a list item applies to your research, read the appropriate section before selecting a response.

Materials & experimental systems

n/a	Involved in the study	
☐	☒	Antibodies
☐	☒	Eukaryotic cell lines
☒	☐	Palaeontology and archaeology
☐	☒	Animals and other organisms
☐	☒	Human research participants
☒	☐	Clinical data
☒	☐	Dual use research of concern

Methods

n/a	Involved in the study	
☒	☐	ChIP-seq
☐	☒	Flow cytometry
☒	☐	MRI-based neuroimaging

Antibodies

Antibodies used

HSPB1 (1:1000, SPA-800, Enzo Life Sciences, Farmingdale, NY, USA)
HSPB2 (1:1000, SAB4501458, Sigma-Aldrich, Saint Louis, MI, USA)
HSPB3 (1:500, ab213591, Abcam, Cambridge, UK)
HSPB4 (1:1000, PA5-72139, Thermo Fisher Scientific, Waltham, MA, USA)
HSPB5/CRYAB (1:500, MAB4849, R&D Systems, Minneapolis, MN, USA)
HSPB6 (1:2500, MAB4200, R&D Systems, Minneapolis, MN, USA)
HSPB7 (1:200, sc-393739, Santa Cruz Biotechnology Inc., Santa Cruz, CA, USA)
HSPB8 (1:1000, #3059, Cell Signaling Technology, Danvers, MA, USA)
HSPB9 (1:1000, PA5-49139, Thermo Fisher Scientific, Waltham, MA, USA)
Hsp90 (1:200, sc-13119, Santa Cruz Biotechnology Inc., Santa Cruz, CA, USA)
BAG3 (1:1000, A302-806A, Bethyl Laboratories Inc., Montgomery, TX, USA)

VDAC1 (1:1000, ab14734, Abcam, Cambridge, UK)
Tubulin (1:10,000, ab7291, Abcam, Cambridge, UK)
TOM20 (1:1000, ab56783, Abcam, Cambridge, UK)
AIFM1 (1:1000, GTX102399, Genetex, Irvine, CA, USA)
AIFM1 (1:1000, #4642, Cell Signaling Technology, Danvers, MA, USA)
HCCS (1:2000, 15118-1-AP, Proteintech Group, Rosemont, IL, USA)

SDHA (1:3000, GTX632636, Genetex, Irvine, CA, USA)
mHSP60 (1:200, sc-13115, Santa Cruz Biotechnology Inc., Santa Cruz, CA, USA)

CytC (1:1000, #4272, Cell Signaling Technology, Danvers, MA, USA)
V5 (1:1000, R96025, Invitrogen, Carlsbad, CA, USA)
GFP (1:1000, ab290, Abcam, Cambridge, UK)
GFP (1:25, ab6556, Abcam. Cambridge, UK)
GFP (1:1000, 1:5000, GTX113617, Genetex, Irvine, CA, USA)
FLAG (1:1000, F7424, Sigma-Aldrich, Saint Louis, MI, USA)

Mic19/CHCHD3 (1:1000, GTX119821, Genetex, Irvine, CA, USA)
MFN2 (1:1000, M6319, Sigma-Aldrich, Saint Louis, MI, USA)
TIM23 (1:200, sc-514463, Santa Cruz Biotechnology Inc., Santa Cruz, CA, USA)
SLC25A11 (1:200, sc-515593, Santa Cruz Biotechnology Inc., Santa Cruz, CA, USA)
SLC25A12 (1:1000, ab200201, Abcam, Cambridge, UK)
SLC25A13 (1:200, sc-393303, Santa Cruz Biotechnology Inc., Santa Cruz, CA, USA)
SLC25A22 (1:1000, SAB2702048, Sigma-Aldrich, Saint Louis, MI, USA)
IMMT/Mitofillin (1:500, GTX115523, Genetex, Irvine, CA, USA)
DNAJC11 (1:1000, 17331-1-AP, Proteintech, Rosemont, IL, USA)
ATP5C1 (1:1000, 60284-1-AP, Proteintech, Rosemont, IL, USA)
UQCRC2 (1:200, sc-390378, Santa Cruz Biotechnology Inc., Santa Cruz, CA, USA)
MAIP1 (1:500 ,24930-1-AP, Proteintech, Rosemont, IL, USA)
ECHS1 (1:200, sc-515270, Santa Cruz Biotechnology Inc., Santa Cruz, CA, USA)
MTHFDL1L (1:200, sc-376722, Santa Cruz Biotechnology Inc., Santa Cruz, CA, USA)

SLP2 (1:1000, 10348-1-AP, Proteintech, Rosemont, IL, USA)
CHCHD4 (1:1000, 21090-1-AP, Proteintech, Rosemont, IL, USA)

YMEL1 (1:1000, 11510-1-AP, Proteintech, Rosemont, IL, USA)
PARL (1:1000, 26679-1-AP, Proteintech, Rosemont, IL, USA)
ClpB (1:1000, A9130, ABclonal, Woburn, MA, USA)

pHSPB1 phospho-ser15 (1:500, A00343, Genscript Corporation, Piscataway, NJ, USA)
pHSPB1 phospho-ser15 (1:1000, ab5581, Abcam, Cambridge, UK)
pHSPB1 phospho-ser78 (1:500, A00528, Genscript Corporation, Piscataway, NJ, USA)
pHSPB1 phospho-ser78 (1:1000, ab32501, Abcam, Cambridge, UK
pHSPB1 phospho-ser82 (1:500, A00530, Genscript Corporation, Piscataway, NJ, USA)
pHSPB1 phospho-ser82 (1:1000, ab155987, Abcam, Cambridge, UK)

anti-Giantin (1:100, sc-46993, Santa Cruz Biotechnology Inc., Santa Cruz, CA, USA)
anti-KDEL (1:100, ADI-SPA-827, Enzo Life Sciences, Farmingdale, NY, USA)

HRP Goat anti-Rabbit (1:10,000, 111-035-144, Jackson ImmunoResearch Laboratories)
HRP Goat anti-Mouse (1:10,000, 115-035-146, Jackson ImmunoResearch Laboratories)

Alexa Fluor 488 Goat anti-Mouse (1:500, A11001, Life Technologies)
Alexa Fluor 488 Goat anti-Mouse G1 (1:500, A21121, Life Technologies)
Alexa Fluor 488 Donkey anti-Mouse (1:500, A21206, Life Technologies)
Alexa Fluor 488 Donkey anti-Goat (1:500, A11055, Life Technologies)
Alexa Fluor 488 Donkey anti-Rabbit (1:500, A21206, Life Technologies)
Alexa Fluor 546 Goat anti-Mouse G1 (1:500, A21123, Life Technologies)
Alexa Fluor 594 Goat anti-Mouse G1 (1:500, A21125, Life Technologies)
Alexa Fluor 647 Goat anti-Mouse G2a (1:500, A21241, Life Technologies)

Protein A gold 10nm (1:25, Department of Cell Biology, University of Utrecht)

Validation

Antibodies were selected based on their use in other publications and/or validation by the manufacturers for their respective application. Where possible we used knockdown or knockout cell lines to validate specificity further. Alternatively, some antibodies were validated by verifying their known response to stress stimuli such as increased expression upon heat shock. A last layer of validation was obtained through cell fractionation experiments in which we separated mitochondria from cytosol, allowing us to verify the specificity of antibodies based on the cellular localization of the target protein.

Eukaryotic cell lines

Policy information about cell lines

Cell line source(s)

All cell lines (HeLa, HEK293T, NSC34, Neuro-2a, SH-SY5Y, COS1, A498,C2C12) were acquired from the American Type Culture Collection (ATCC). Primary human-derived lines (lymphoblasts and fibroblasts) were obtained from healthy volunteers according to protocols approved by the local Medical Ethics Committee (University of Antwerp, Belgium). Tafazzin knockout HEK293 Flp-In cells were generously shared by Steven Claypool (The Johns Hopkins University School of Medicine, USA). The origina HEK293 Flp-In cells were obtained from Invitrogen.

Authentication

Authentication was performed upon first arrival in the lab (through morphological and STR analysis).

Mycoplasma contamination	All cell lines were routinely tested for mycoplasma contamination. All cell lines were negative.
Commonly misidentified lines (See ICLAC register)	The cell lines used in this study are not listed as commonly misidentified cell lines. This was verified in the ICLAC table of commonly misidentified cell lines (version 11).

Animals and other organisms

Policy information about studies involving animals; ARRIVE guidelines recommended for reporting animal research

Laboratory animals	Mouse C57BL6/J (Female - 6 months old)
Wild animals	This study did not involve wild animals.
Field-collected samples	This study did not involve field-collected samples.
Ethics oversight	Animal procedures complied with all relevant ethical regulations and were carried out in accordance with European, national and institutional guidelines and with approval by the Medical Ethics Committee (20/36/461) and Ethical Committee for Animal Experiments (2022-13) of the University of Antwerp, Belgium.

Note that full information on the approval of the study protocol must also be provided in the manuscript.

Human research participants

Policy information about studies involving human research participants

Population characteristics	Whole blood was collected from healthy donor (female) for generation of lymphoblast cell lines. Fibroblast cell lines were established from punch biopsies of healthy donor (male).
Recruitment	Donors were recruited as volunteers and received no remuneration. These are adult volunteers interested to contribute to scientific research, and have been recruited on free will.
Ethics oversight	Blood and skin collection was performed according to the guidelines of the the local Medical Ethics Committee (University of Antwerp, Belgium). Healthy donors are anonymous individuals who signed an informed consent prior to blood/skin collection for research use only and at no cost.

Note that full information on the approval of the study protocol must also be provided in the manuscript.

Flow Cytometry

Plots

Confirm that:

☒ The axis labels state the marker and fluorochrome used (e.g. CD4-FITC).

☒ The axis scales are clearly visible. Include numbers along axes only for bottom left plot of group (a 'group' is an analysis of identical markers).

☒ All plots are contour plots with outliers or pseudocolor plots.

☒ A numerical value for number of cells or percentage (with statistics) is provided.

Methodology

Sample preparation	HeLa cells were loaded with 100nM TMRE (tetramethylrhodamine ethyl ester perchlorate, ENZ-52309, Enzo Life Sciences, Farmingdale, NY, USA) for 30 min at 37°C. Cells were washed in PBS, trypsinized and single cells were resuspended in PBS with 2% (v/v) fetal bovine serum prior to analysis. Flow cytometry was performed on a LSR Fortessa cytometer (BD Biosciences). Data analysis was performed with FlowJo10 software (Tree Star Inc., Ashland, OR, USA).
Instrument	LSR Fortessa cytometer (BD Biosciences)
Software	FlowJo10 software (Tree Star Inc., Ashland, OR, USA)
Cell population abundance	All cells were included except those that were not viable.
Gating strategy	Gating was optimized for wild type control HeLa cells and subsequently applied to the sHSP-depleted lines using the same parameter settings. Viable singlet cells were analyzed and separated from potential dead cells or doublets based on scatter.

☒ Tick this box to confirm that a figure exemplifying the gating strategy is provided in the Supplementary Information.
