# Supplementary figures and images for: Small heat shock proteins operate as molecular chaperones in the mitochondrial intermembrane space
Source: Nat Cell Biol. 2023 Jan 23;25(3):467–80. doi: 10.1038/s41556-022-01074-9 (PMC10014586; doi:10.1038/s41556-022-01074-9)

Source data files (Figure 1)

Figure 1a (HeLa)

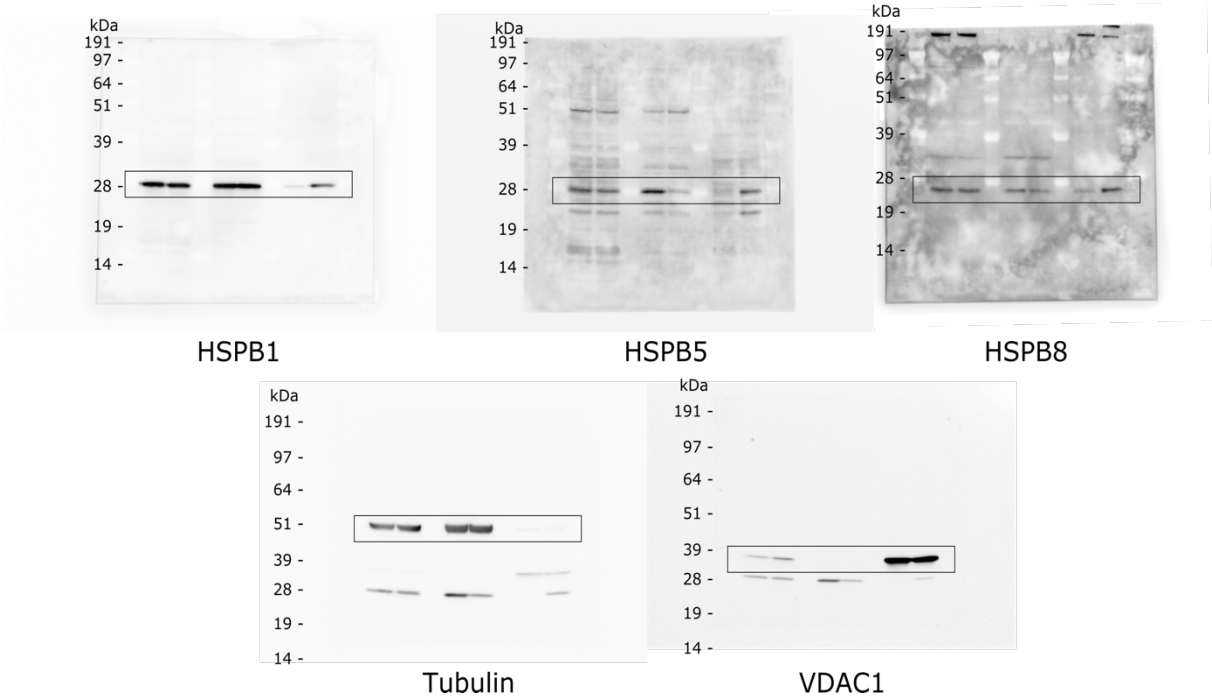

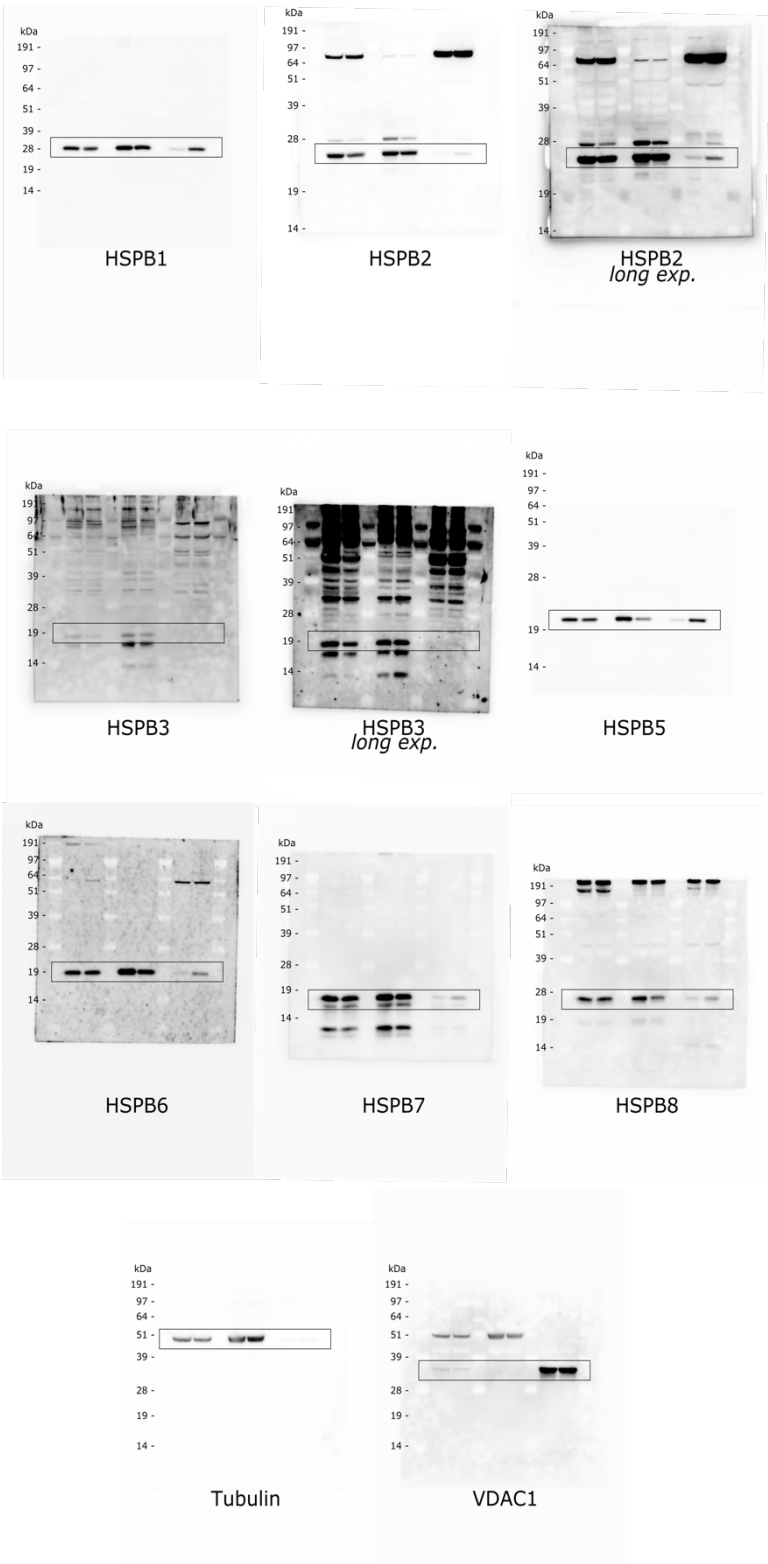

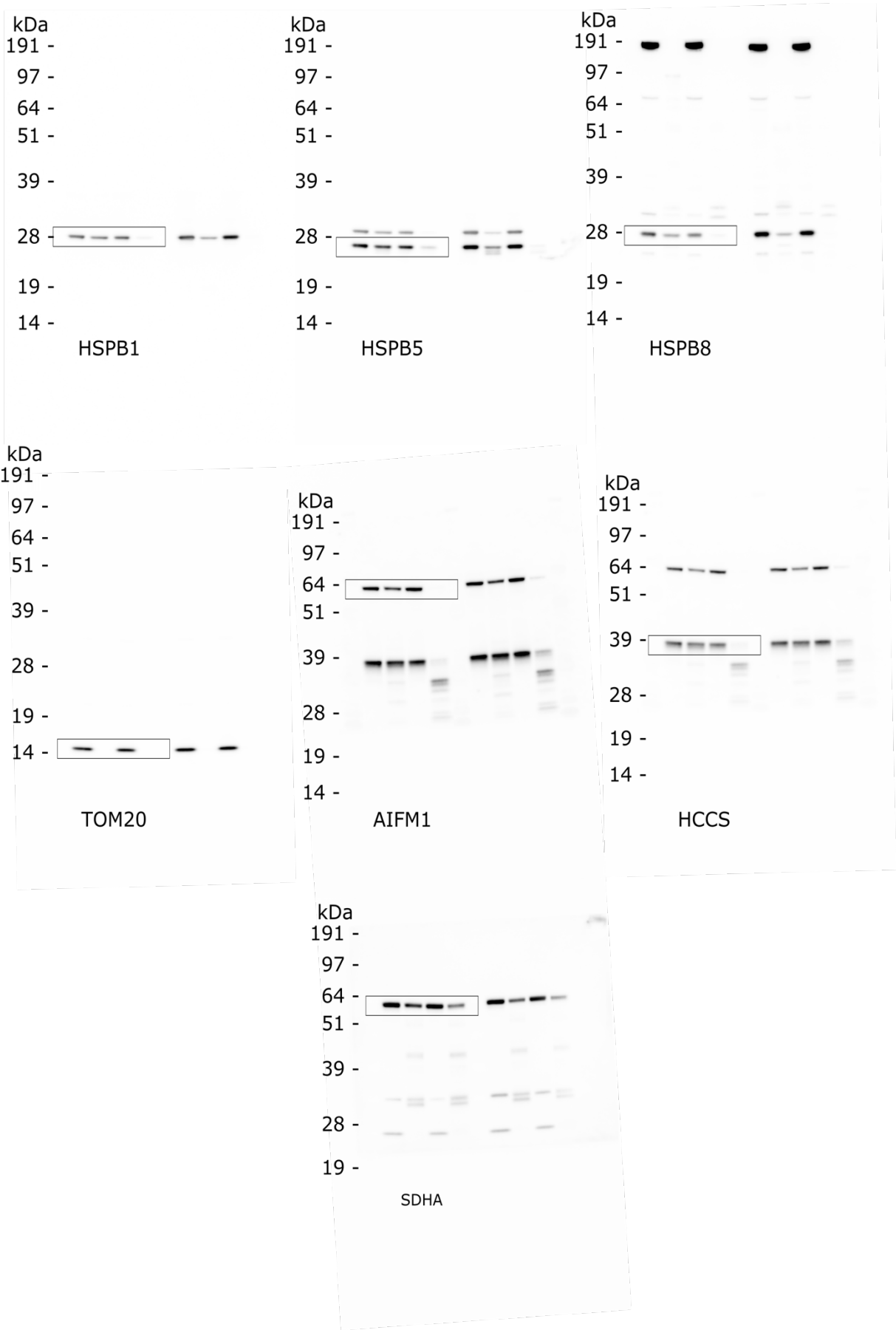

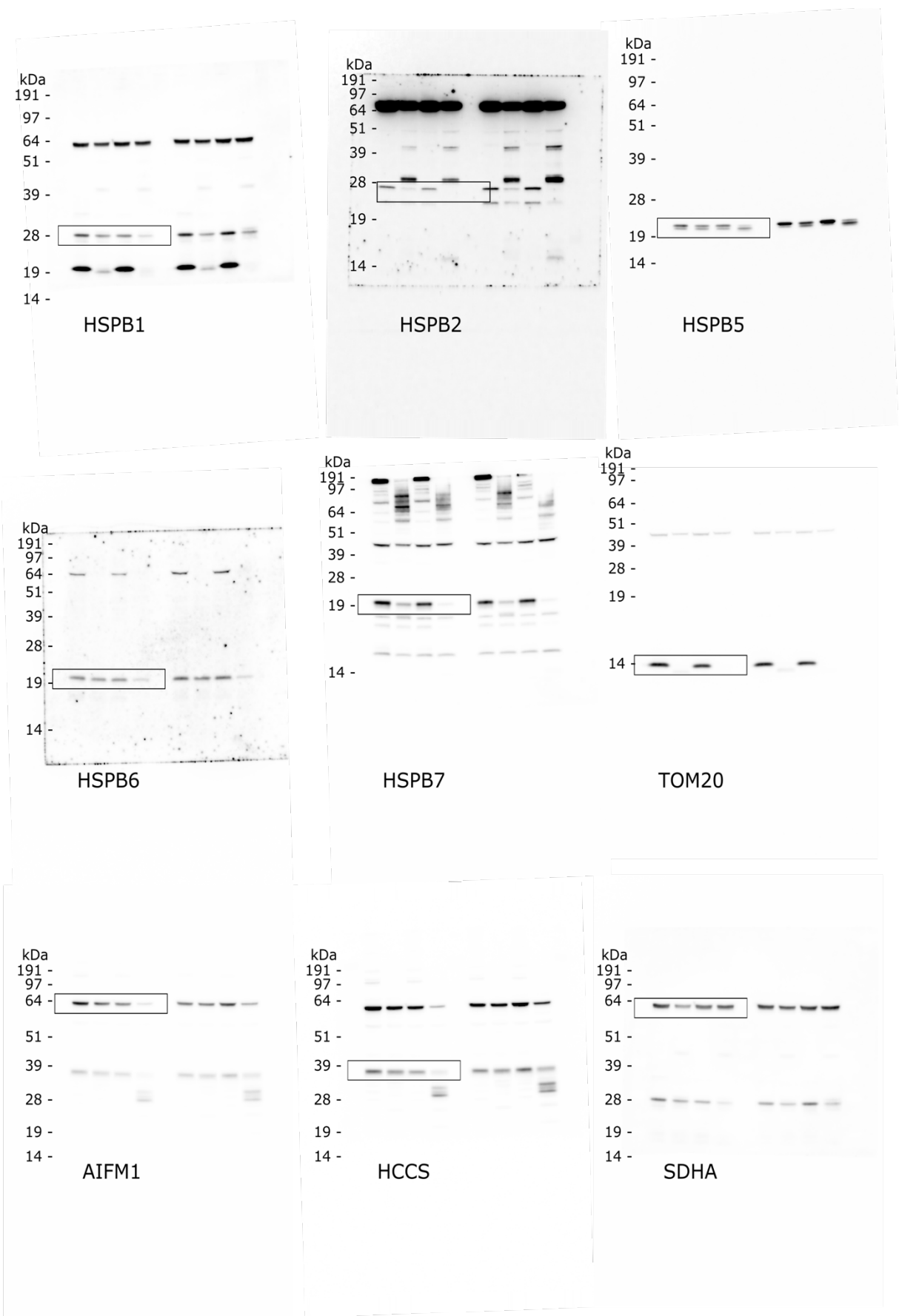

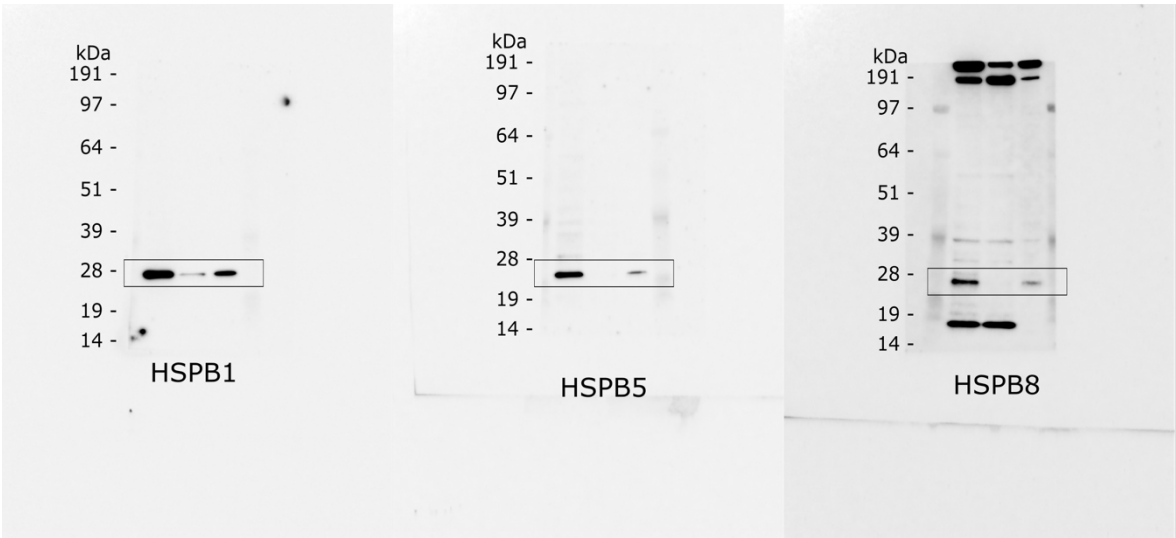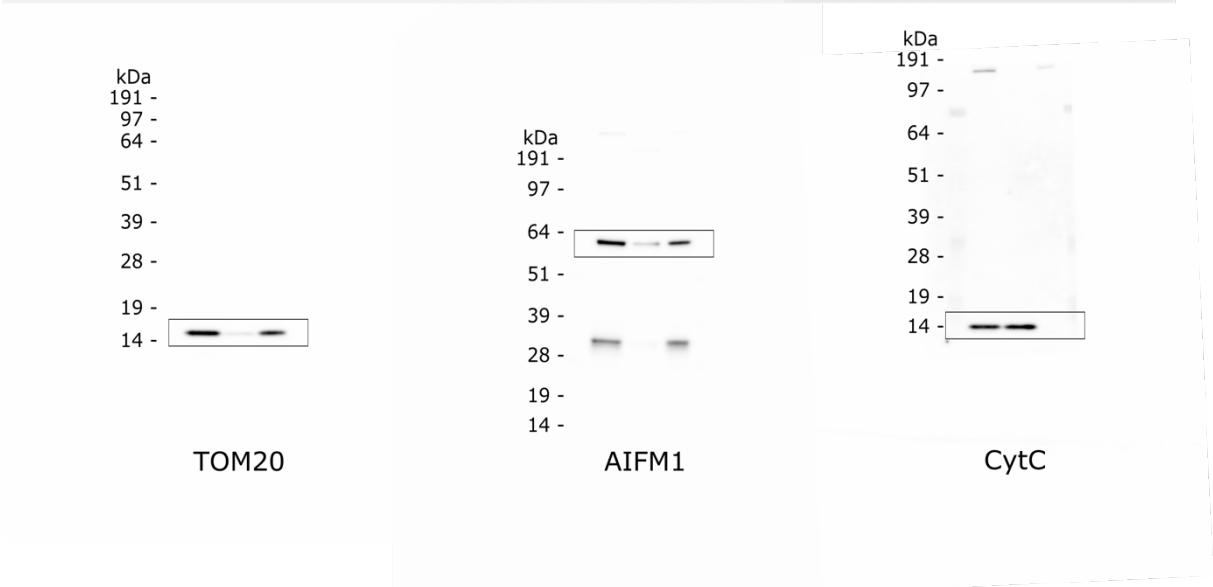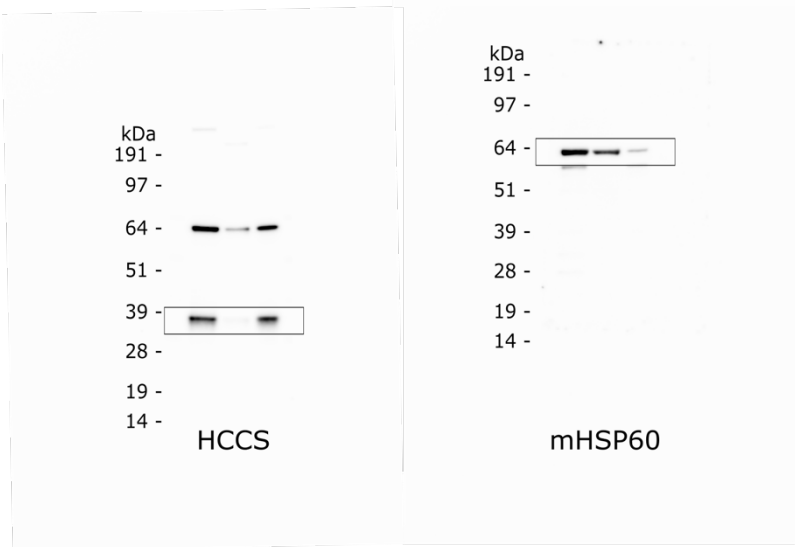

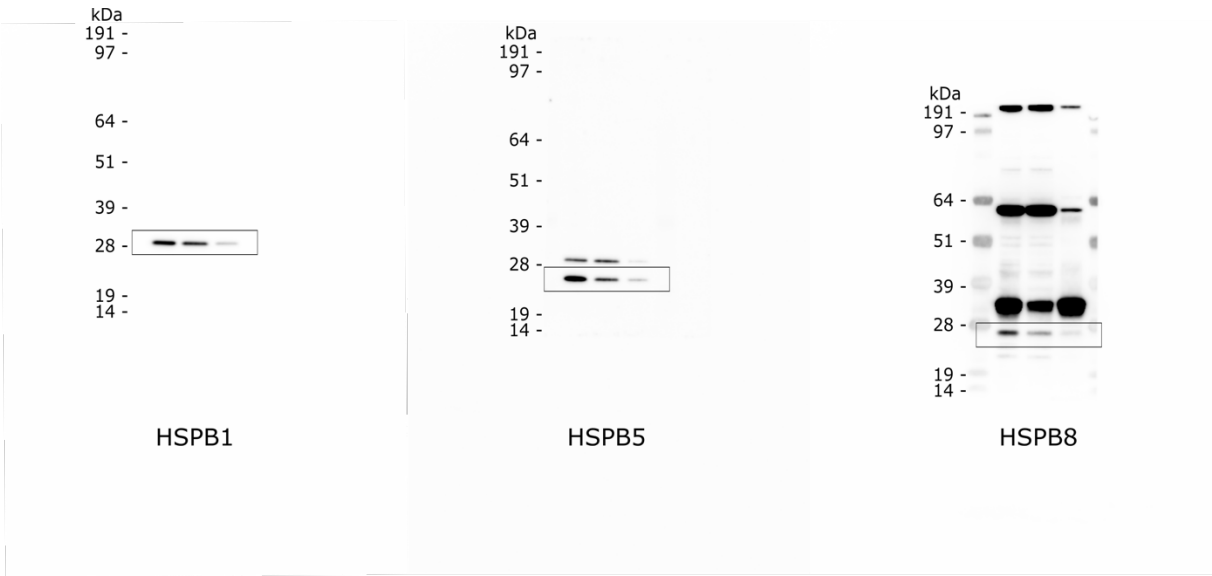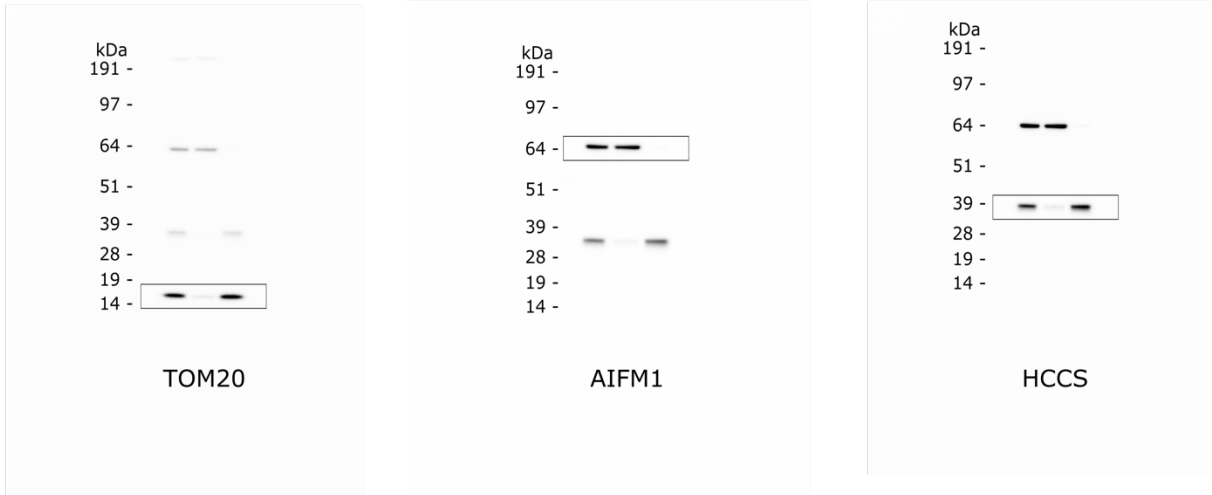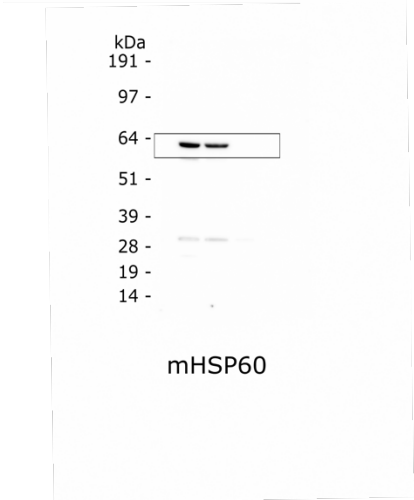

**Figure 1g**

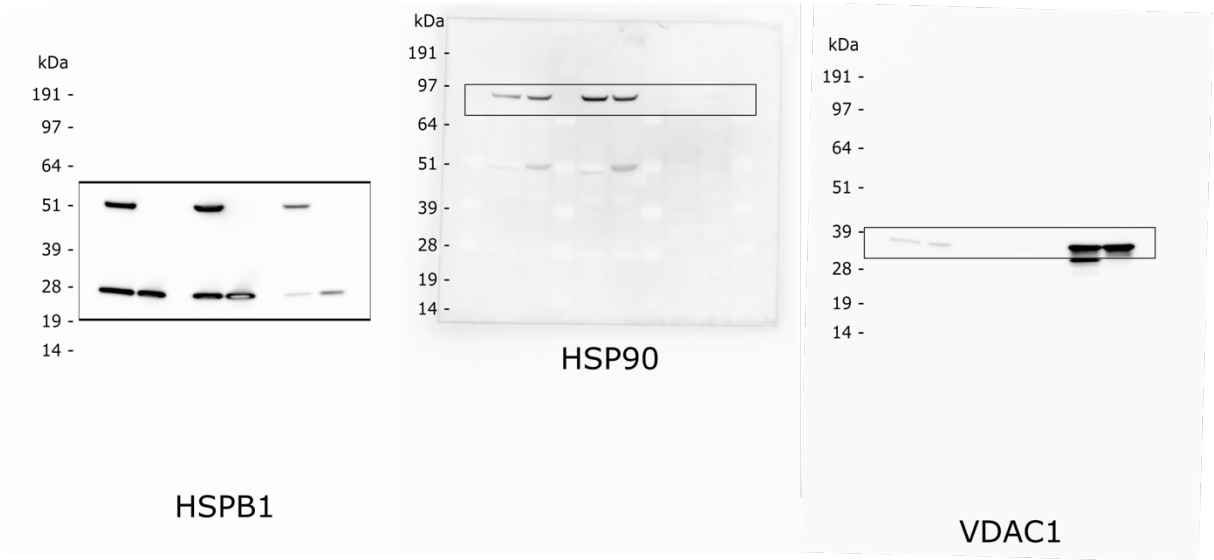

Supplement: Source Data Fig. 1 — Unprocessed western blots. [file 41556_2022_1074_MOESM3_ESM.pdf]

Source data files (Figure 2)

Figure 2a

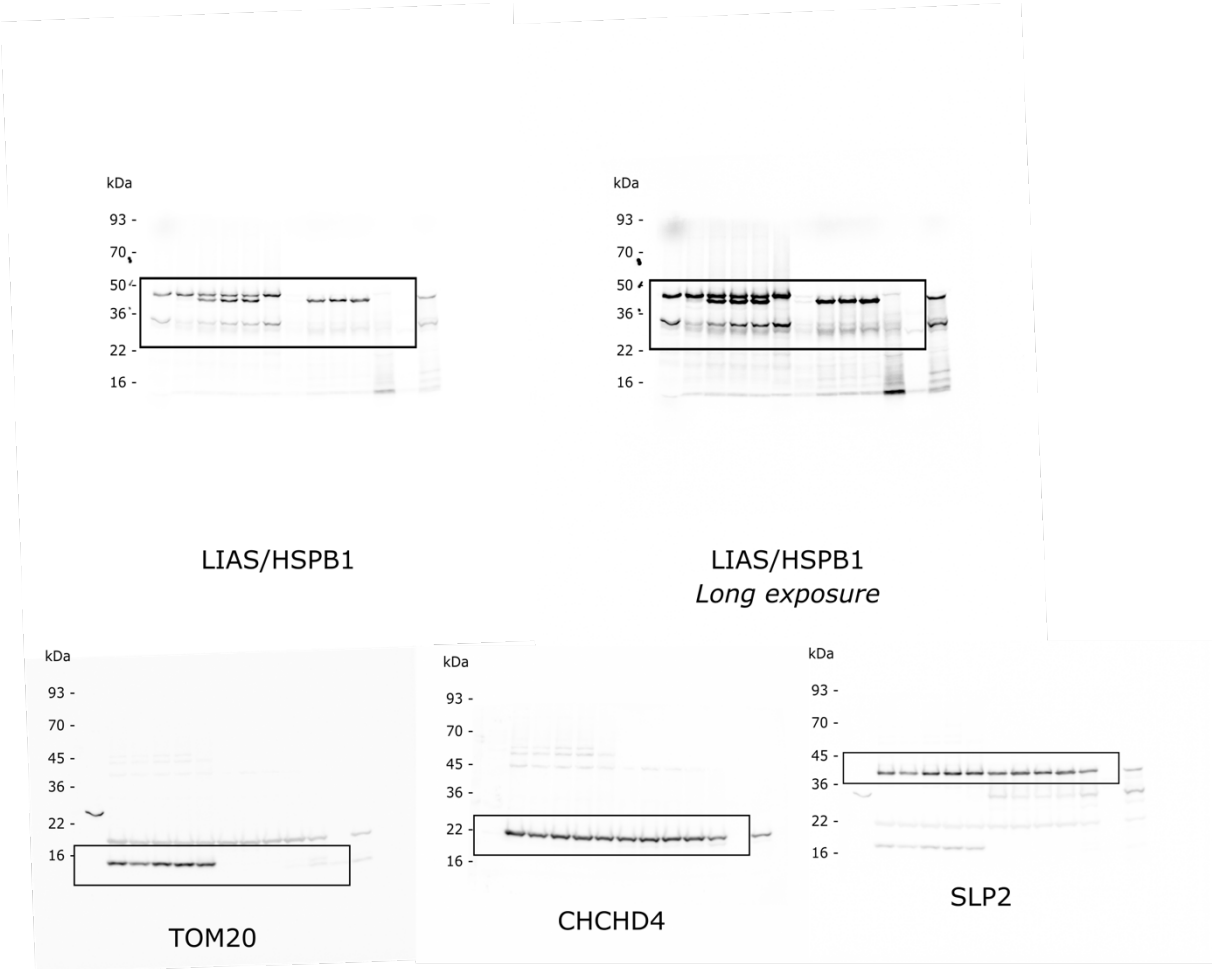

14 **Figure 2c**

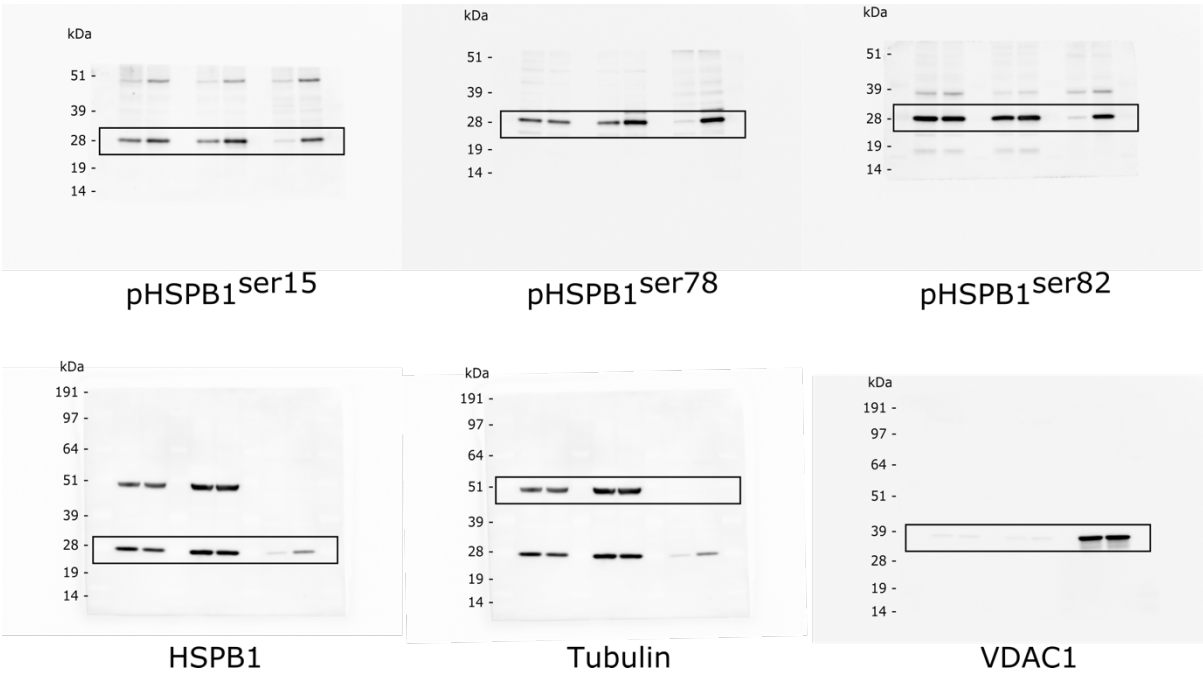

15  
16  
17  
18  
19  
20  
21  
22  
23  
24  
25  
26  
27  
28  
29  
30

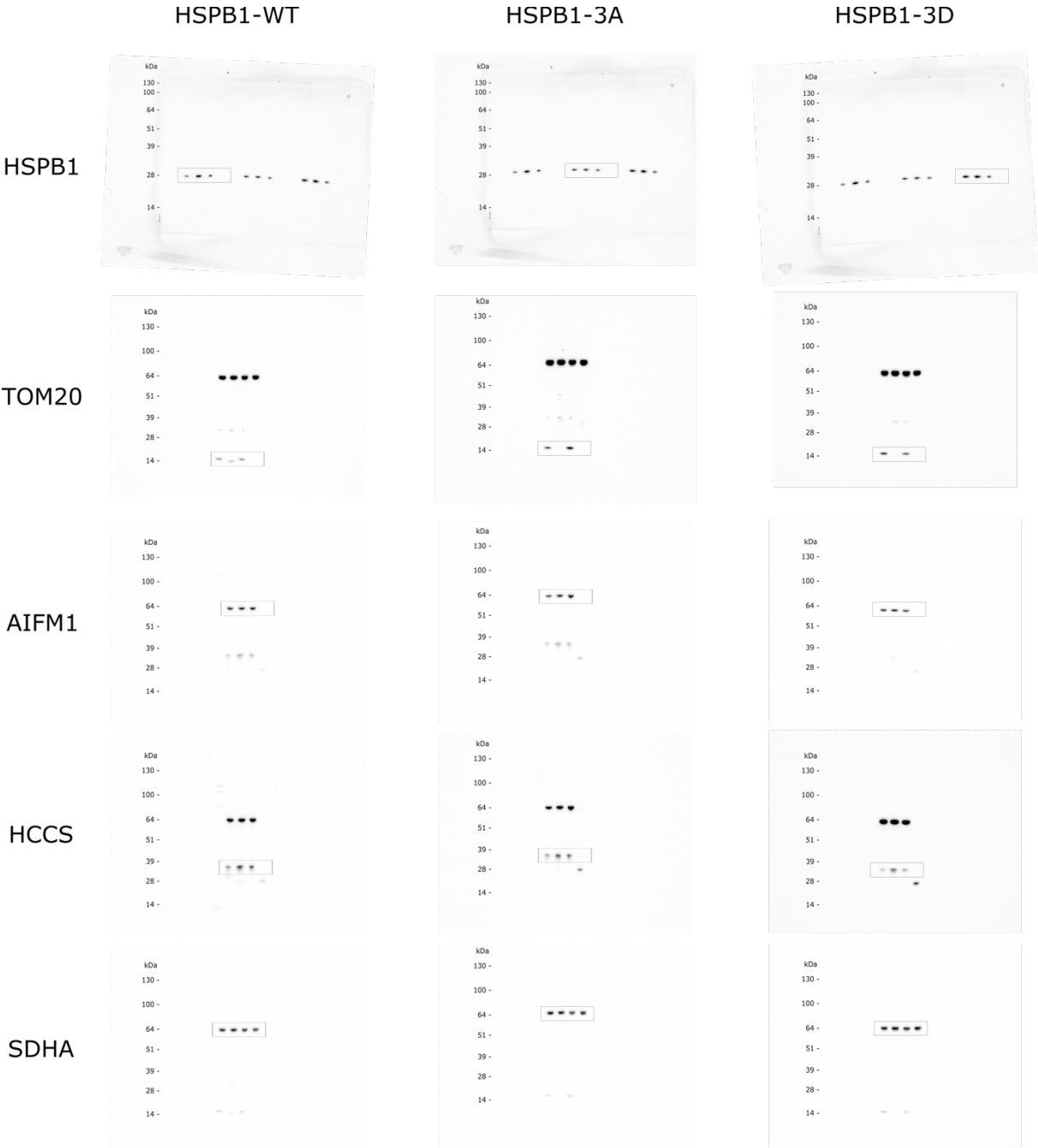

32  
33  
34  
35  
36  
37  
38

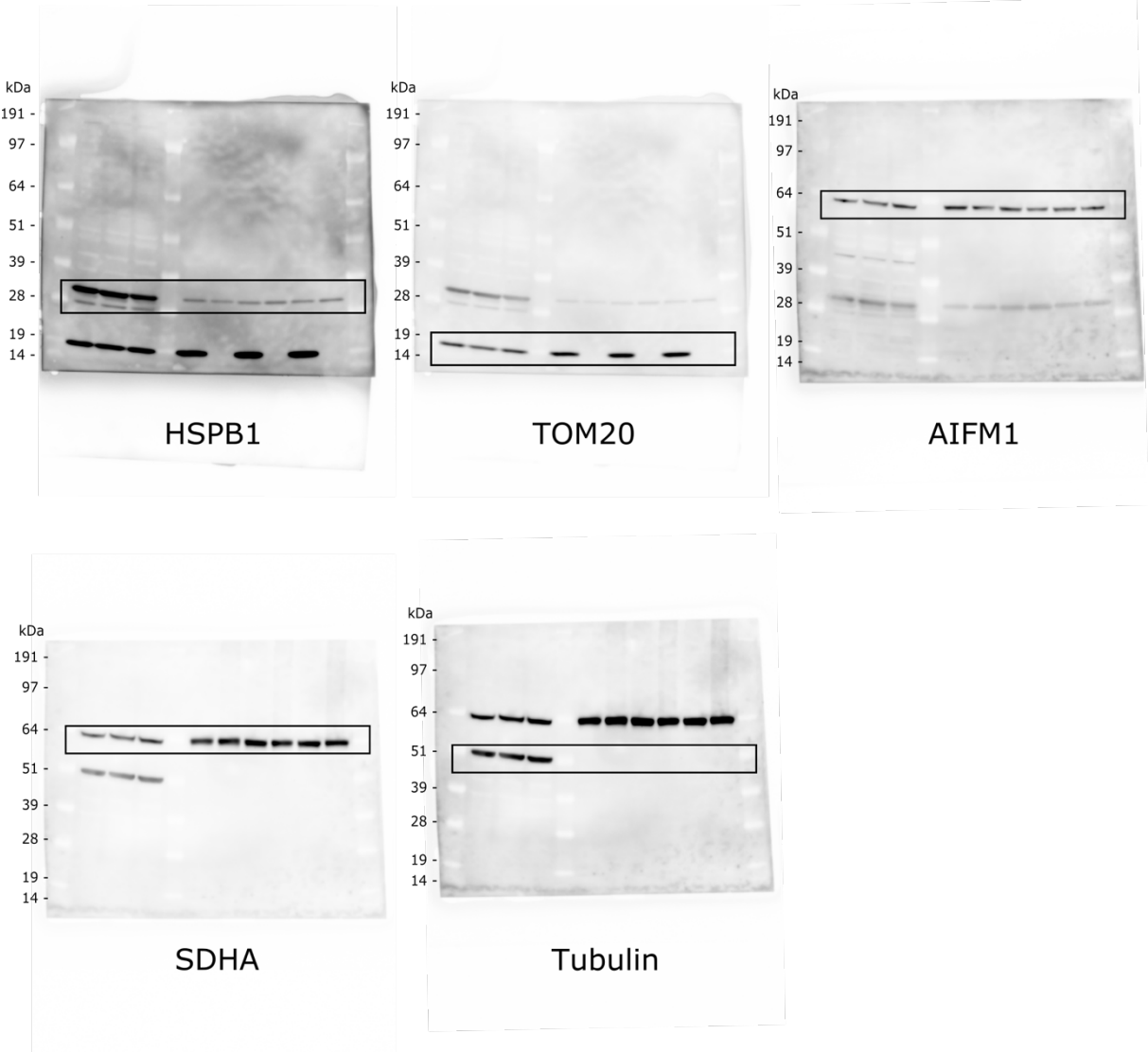

40  
41  
42  
43  
44  
45  
46  
47  
48  
49

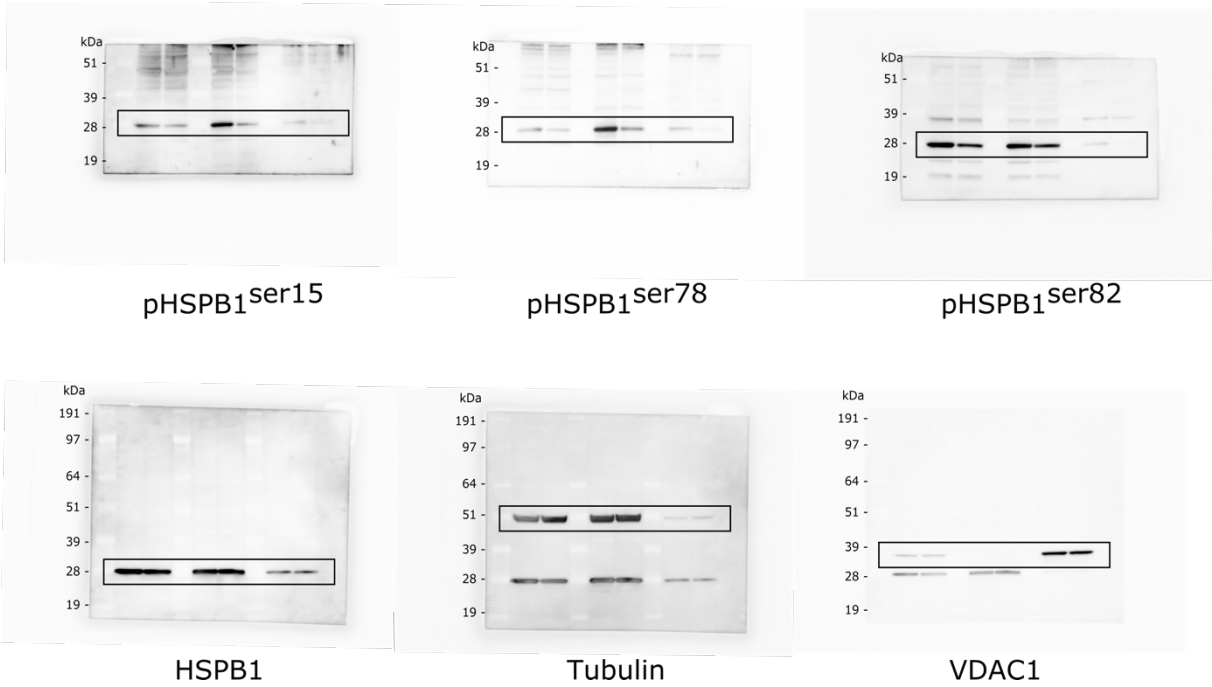

51

52

Supplement: Source Data Fig. 2 — Unprocessed western blots. [file 41556_2022_1074_MOESM5_ESM.pdf]

1

Source data files (Figure 3)

2

3 **Figure 3a**

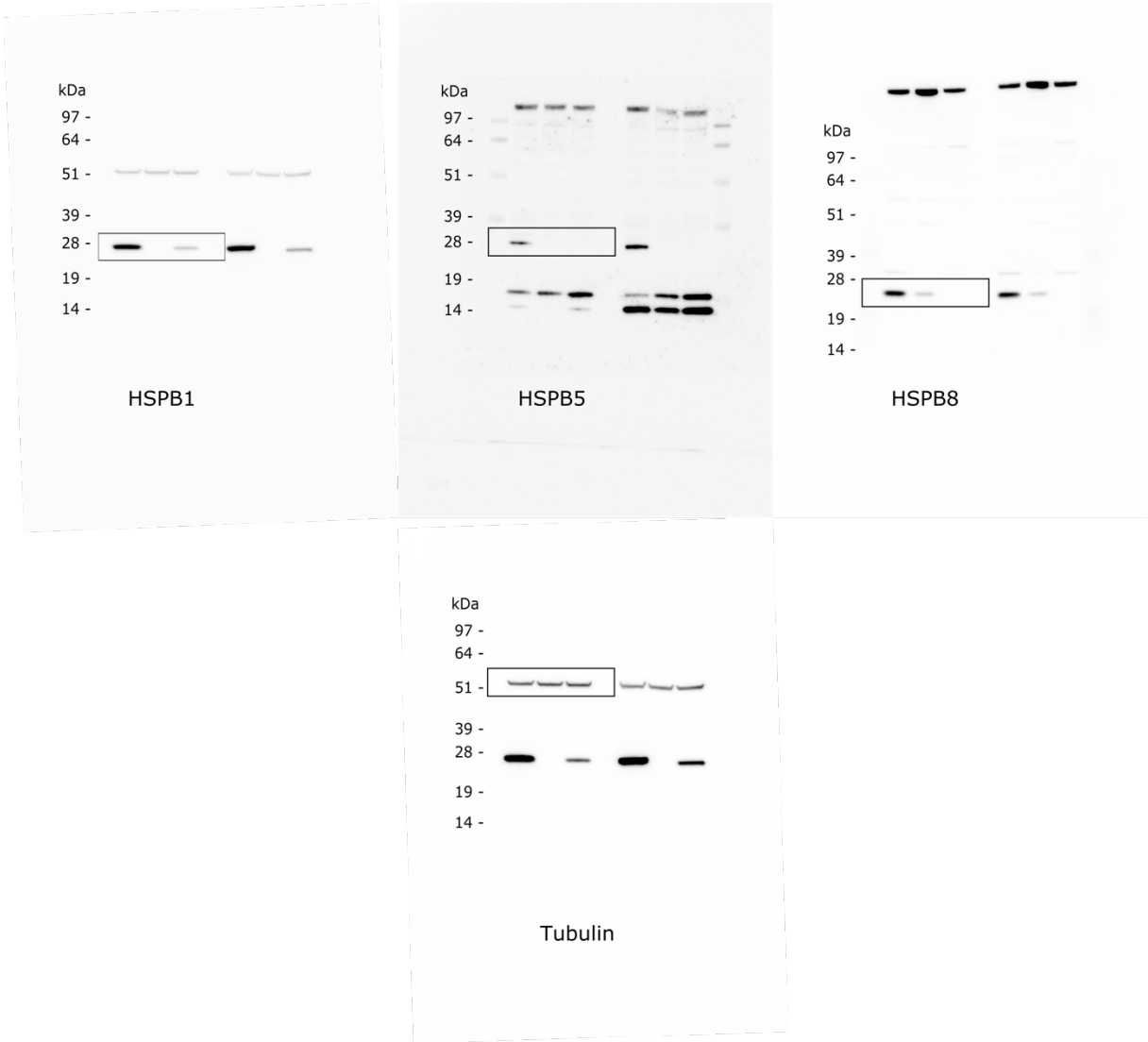

4

Supplement: Source Data Fig. 3 — Unprocessed western blots. [file 41556_2022_1074_MOESM7_ESM.pdf]

Source data files (Figure 4)

Figure 4a

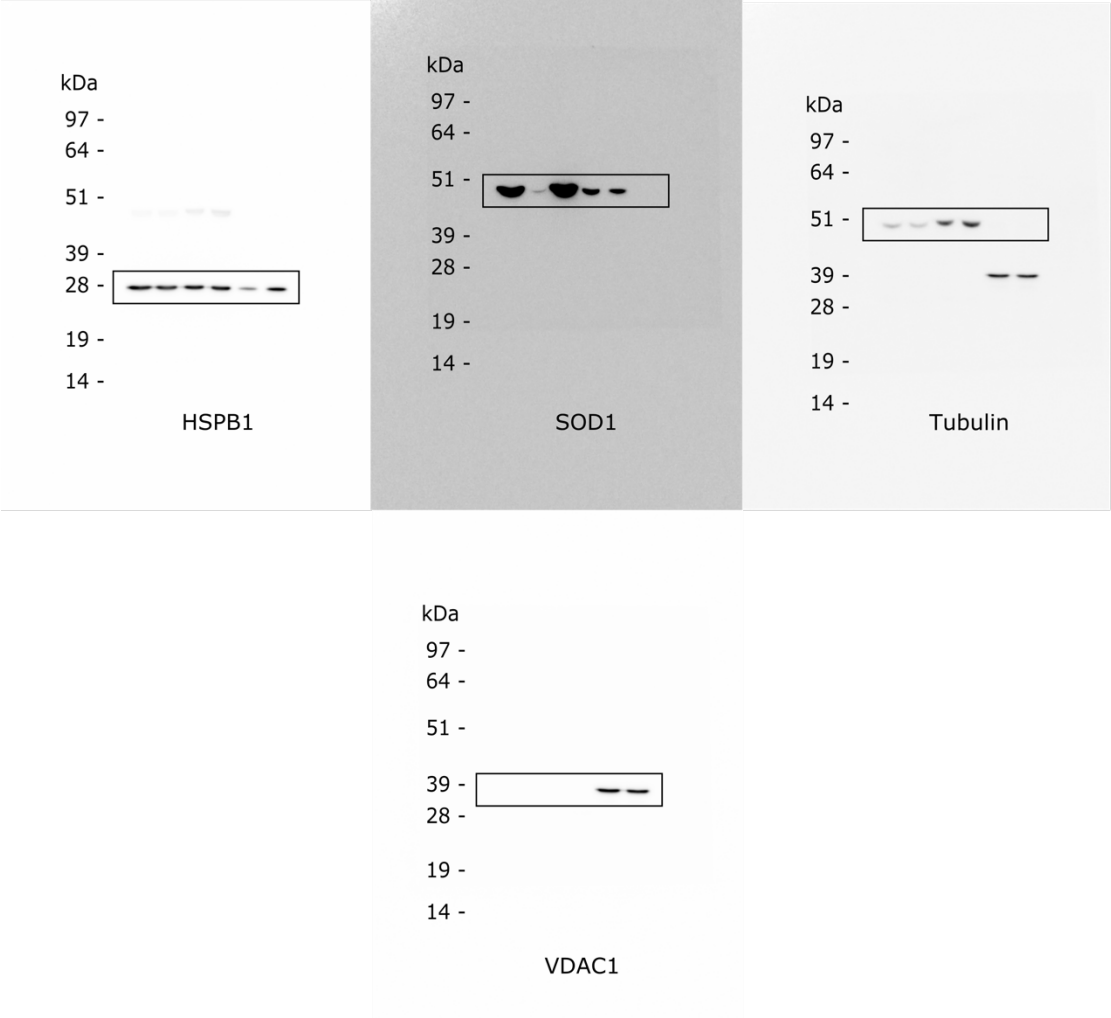

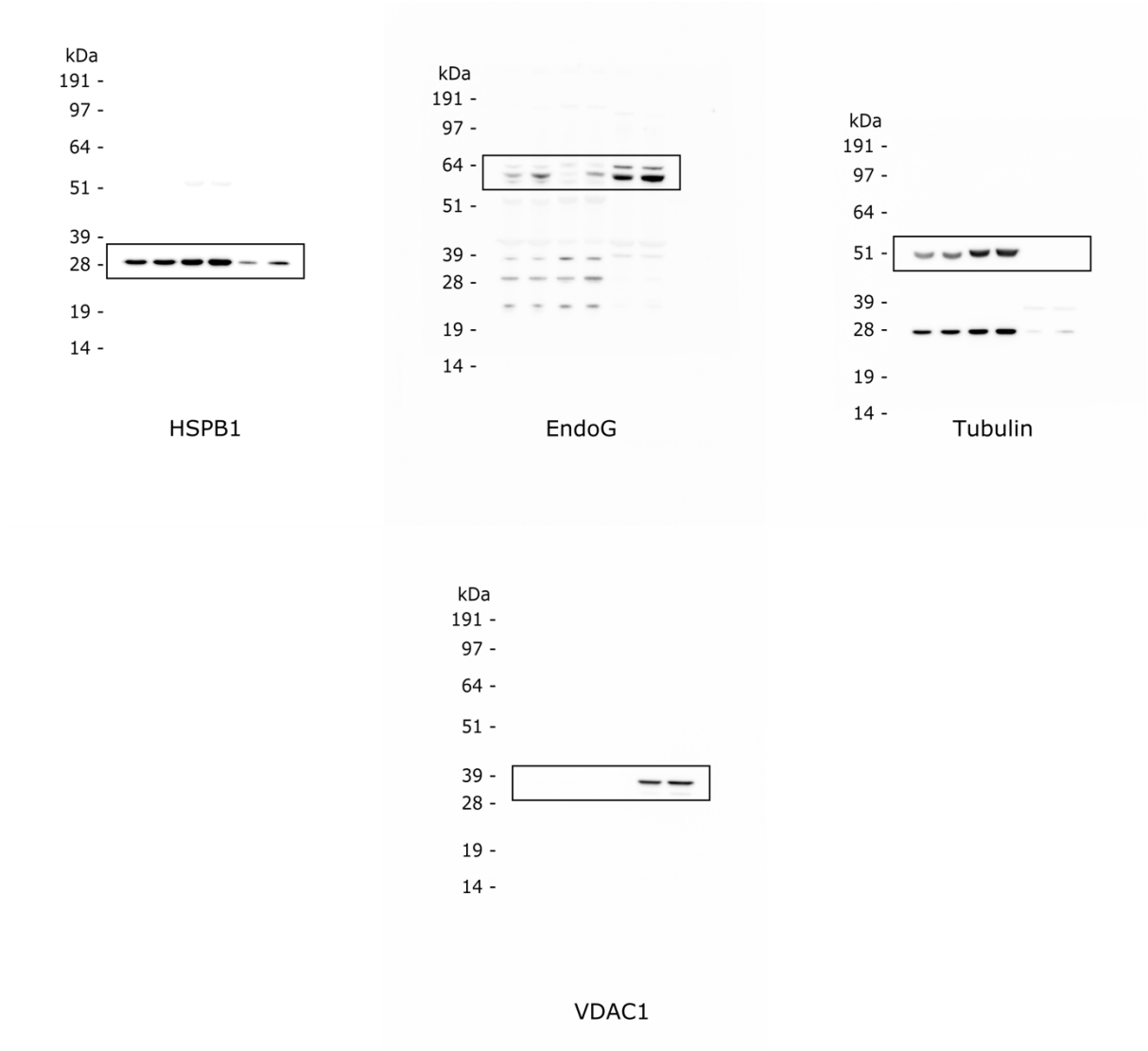

14  
15  
16  
17  
18  
19  
20  
21  
22  
23

24 **Figure 4c**

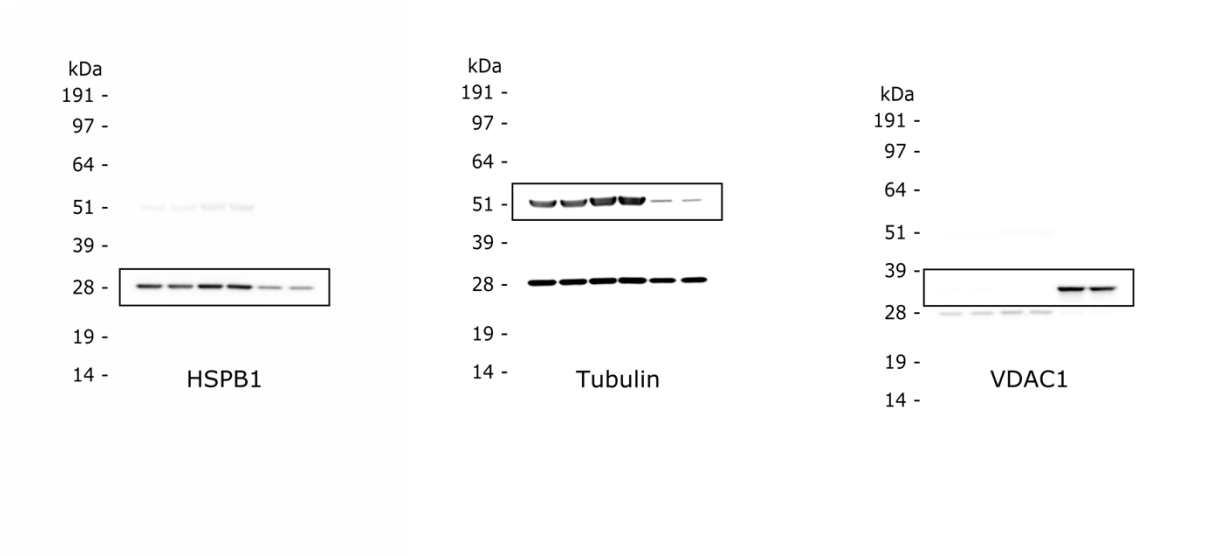

25

26

27

28

29

30

31

32

33

34

35

36

37

38

39

40

41

42

43 **Figure 4d**

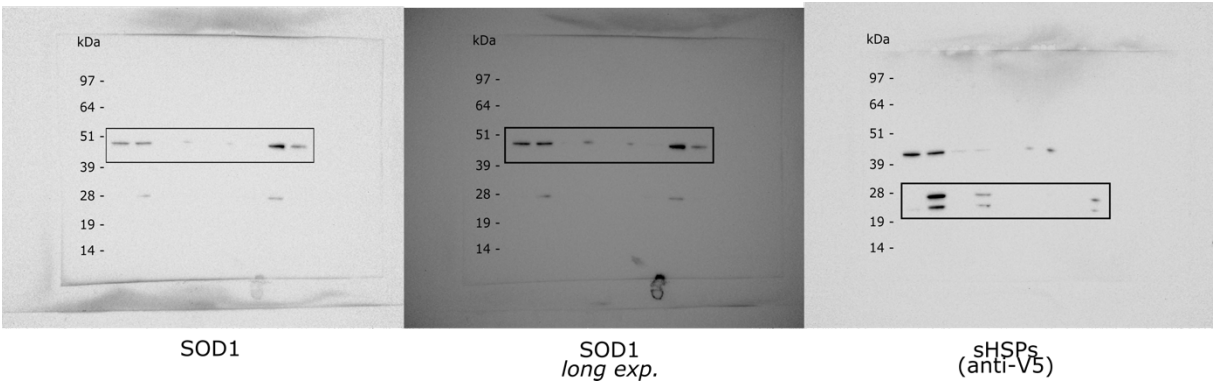

44  
45  
46  
47  
48  
49  
50  
51  
52  
53  
54  
55  
56  
57  
58  
59  
60  
61  
62  
63

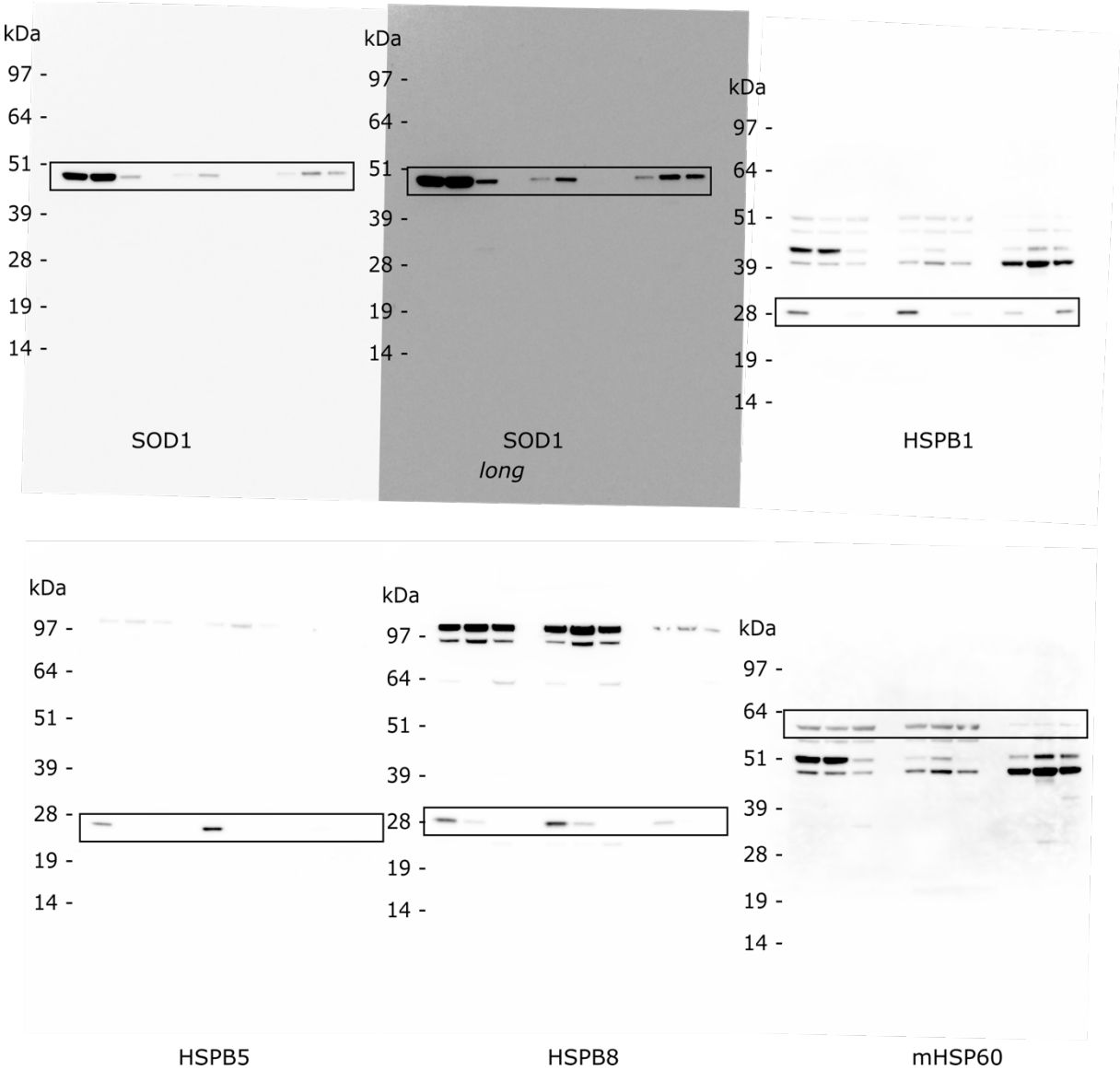

65 HSPB5 HSPB8 mHSP60

66

67

68

69

70

71

72

73

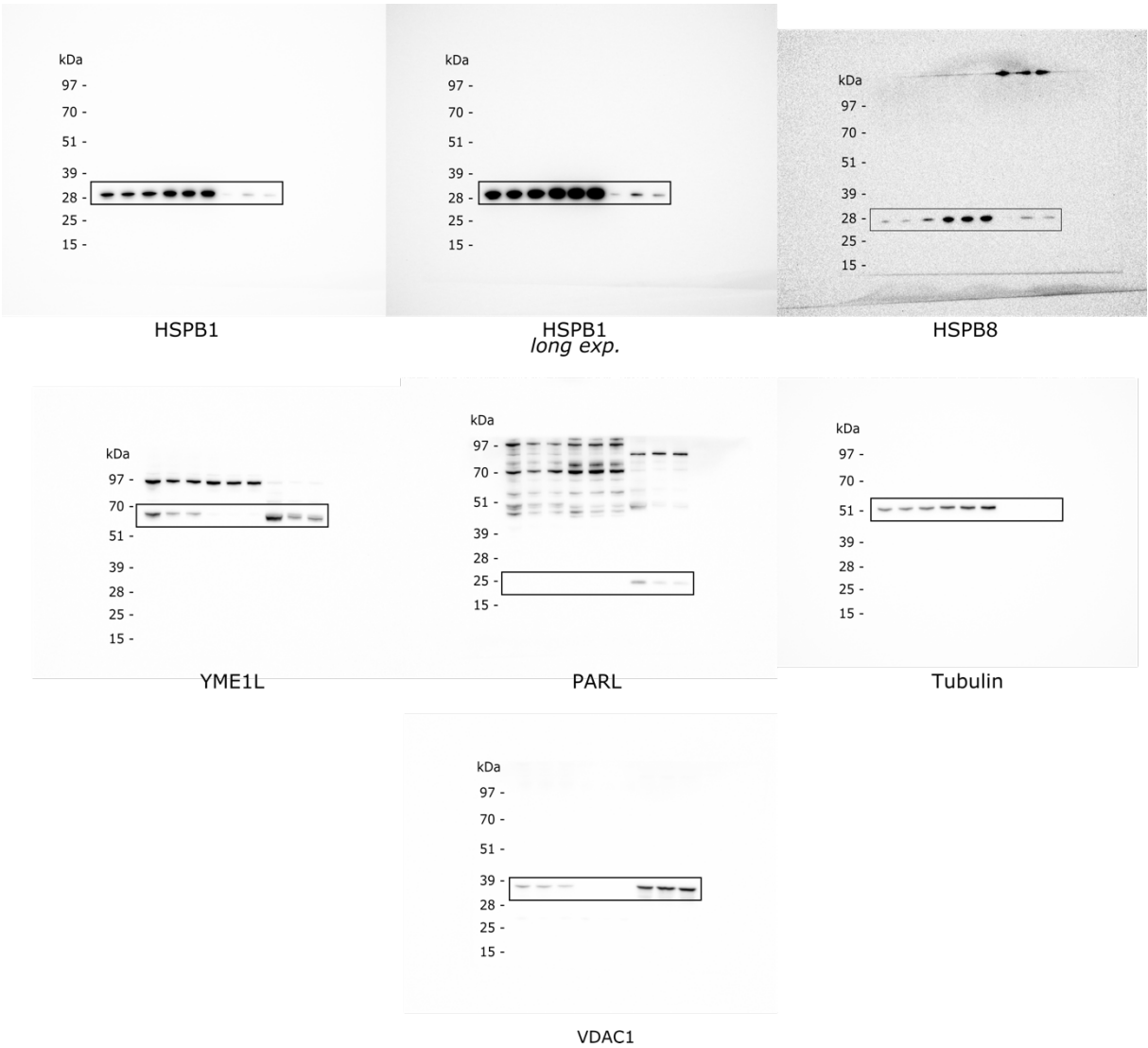

75

76

77

78

79

80

81

82

83

84

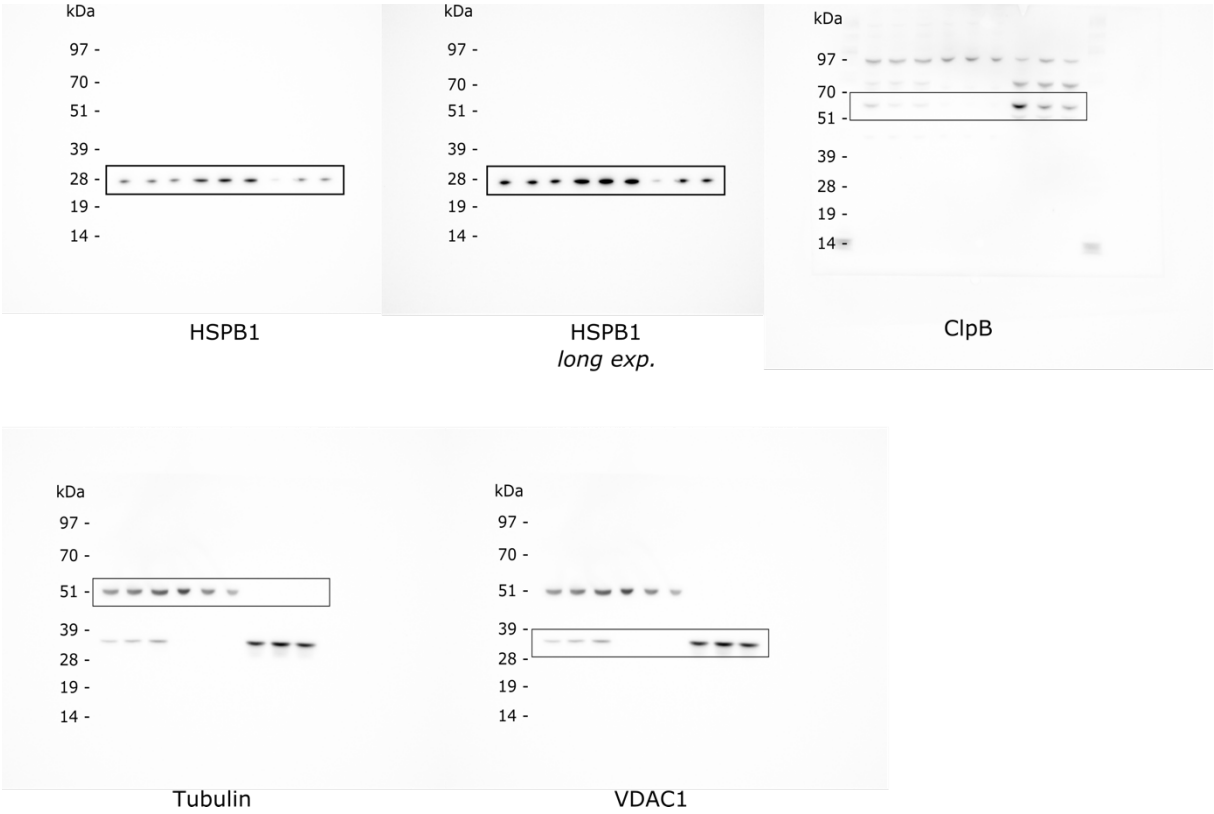

Supplement: Source Data Fig. 4 — Unprocessed western blots. [file 41556_2022_1074_MOESM9_ESM.pdf]

1

Source data files (Figure 5)

2    **Figure 5b**

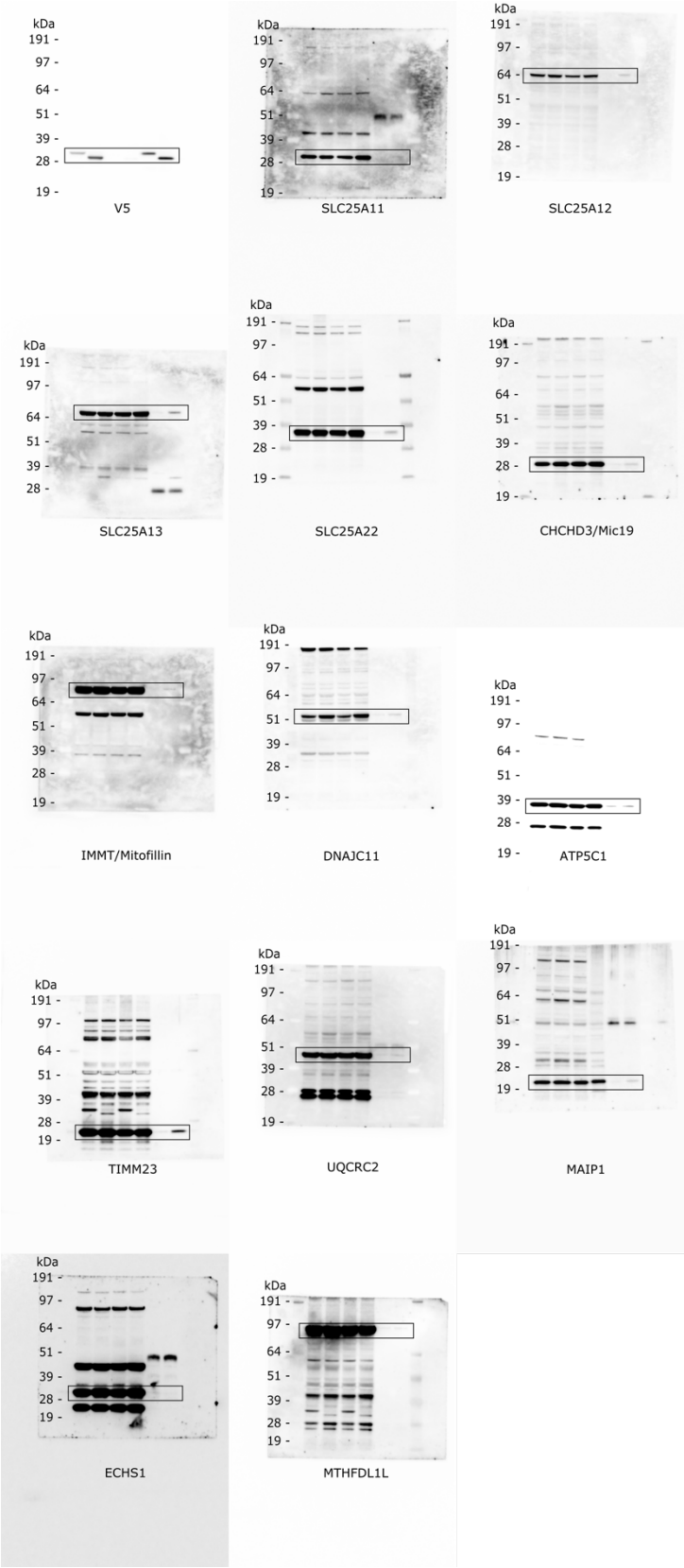

3

4 **Figure 5c**

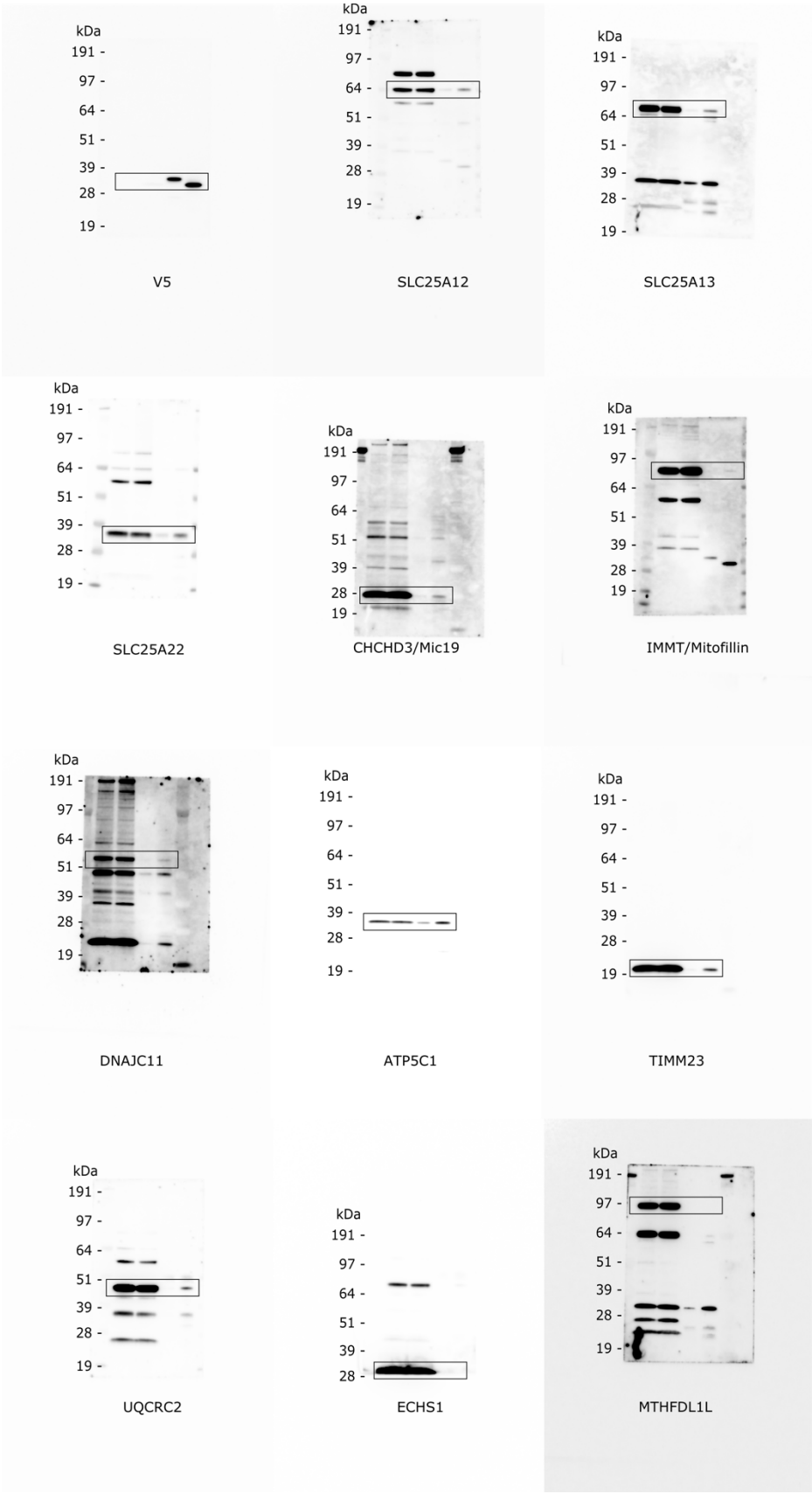

5

6

7 **Figure 5d**

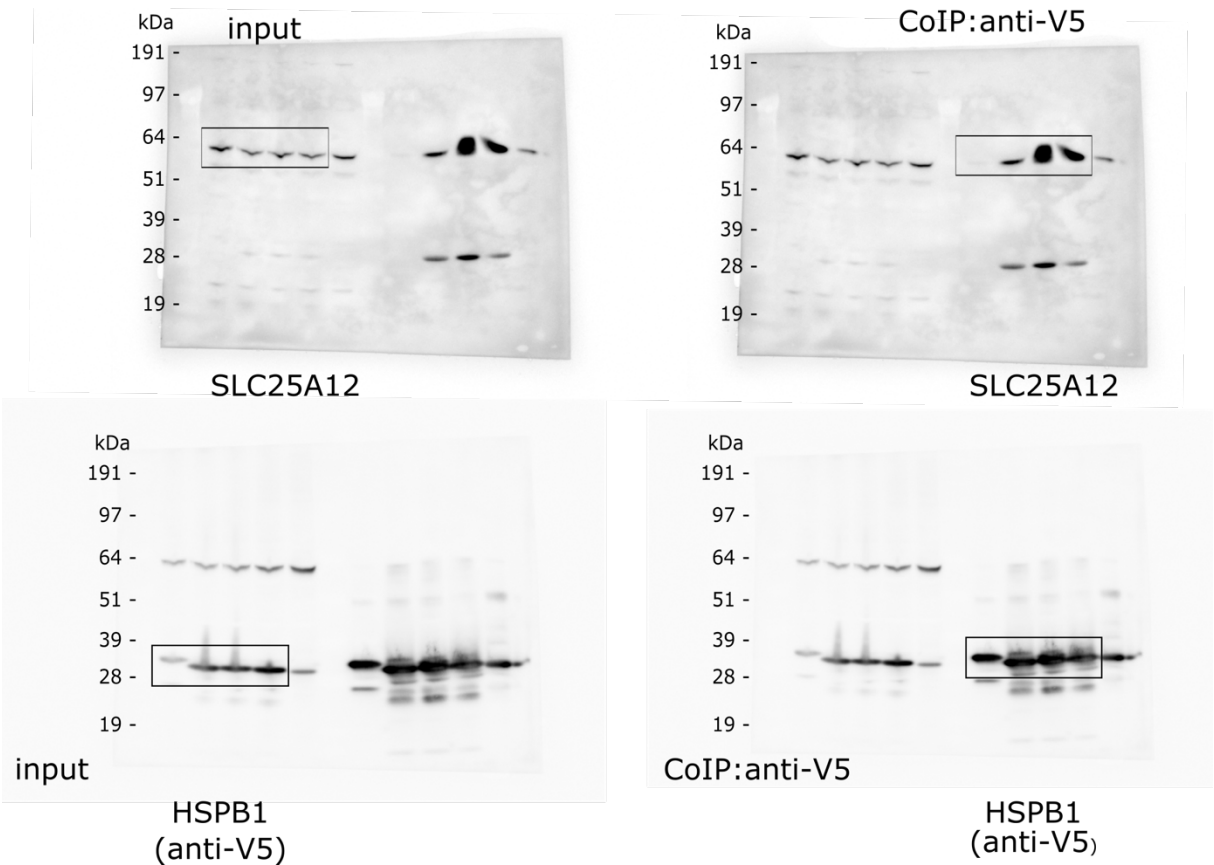

8

9

10

11

12

13

14

15

16

17

18

19

20

21

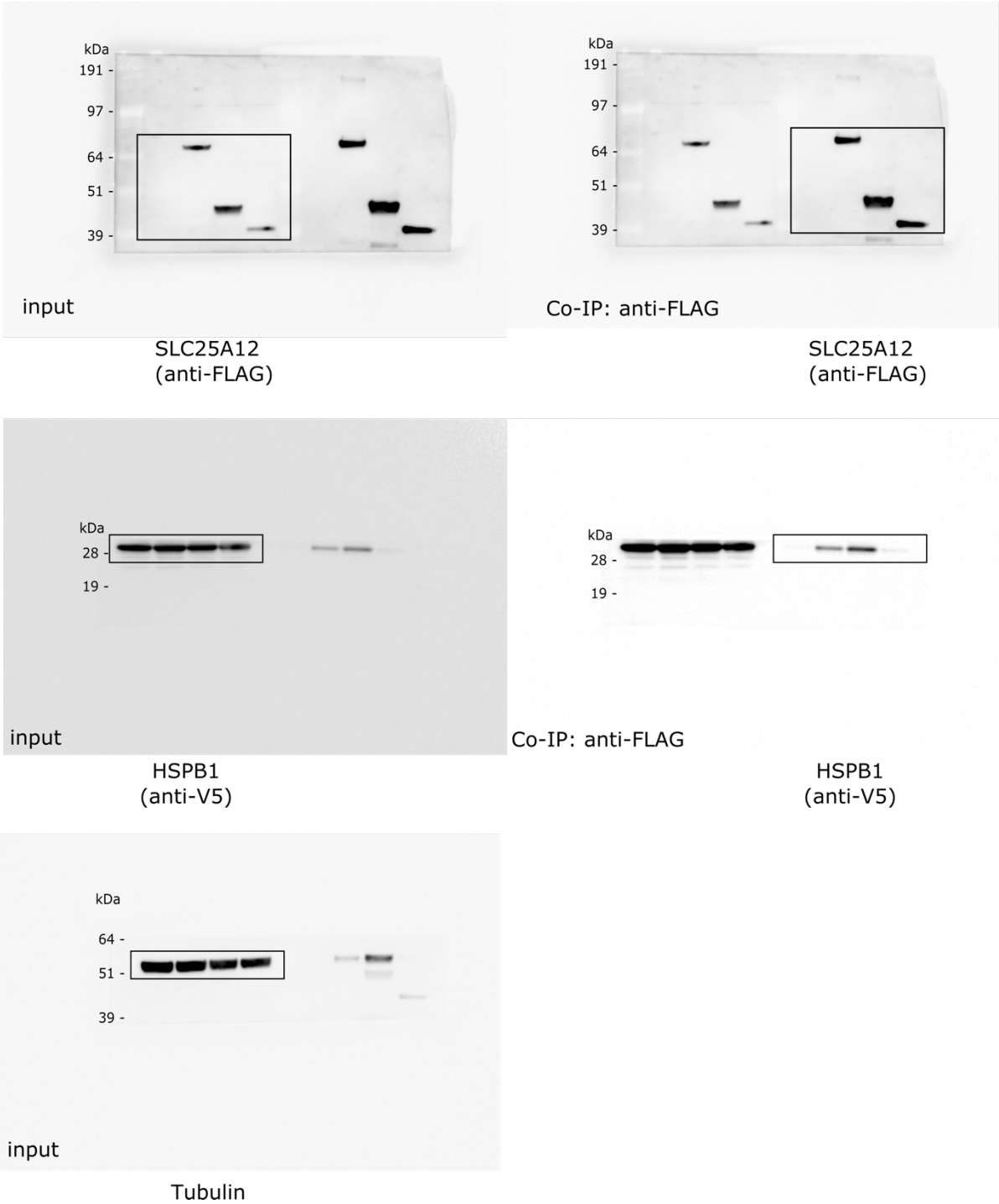

23

24

25

26

27

28

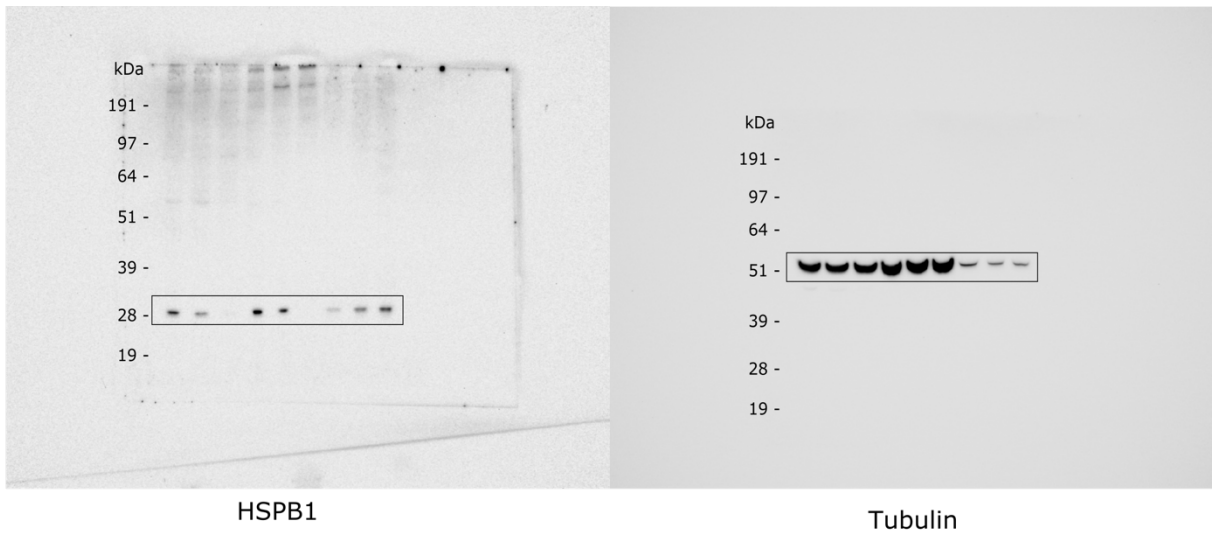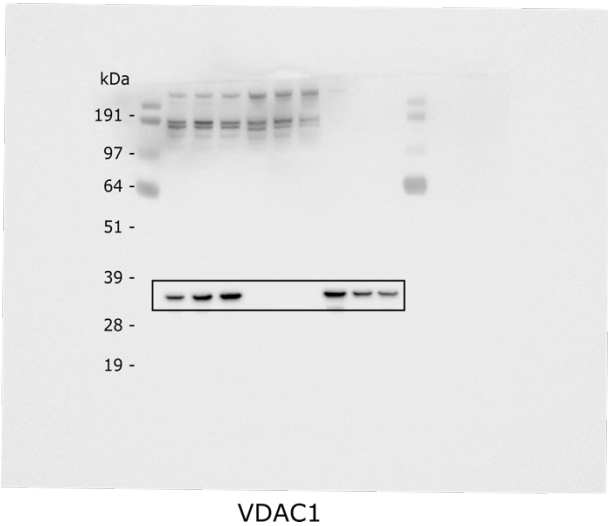

30  
31  
32  
33  
34  
35  
36  
37  
38

Supplement: Source Data Fig. 5 — Unprocessed western blots. [file 41556_2022_1074_MOESM11_ESM.pdf]

Source data files (Figure 6)

Figure 6a

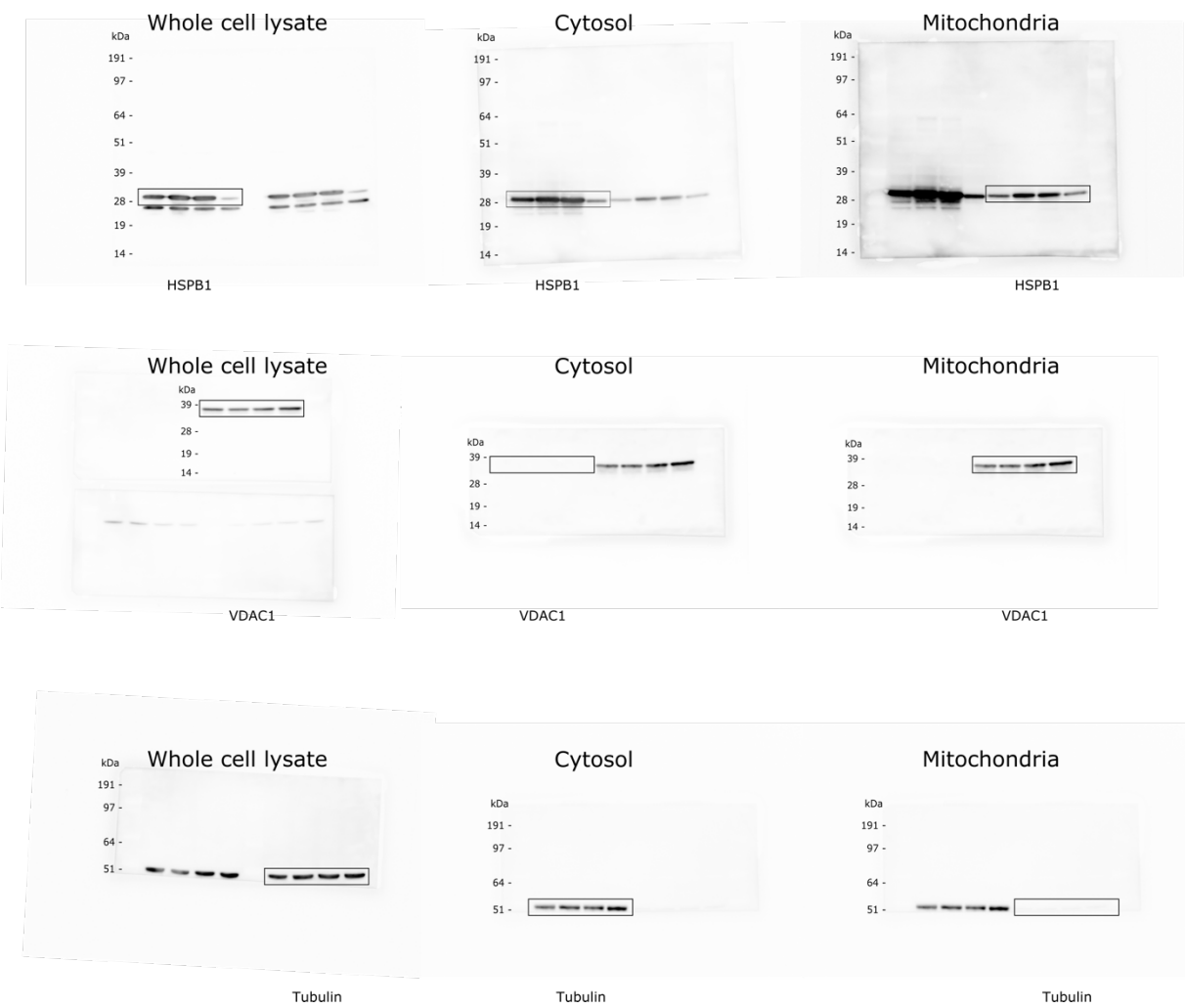

**Figure 6b**

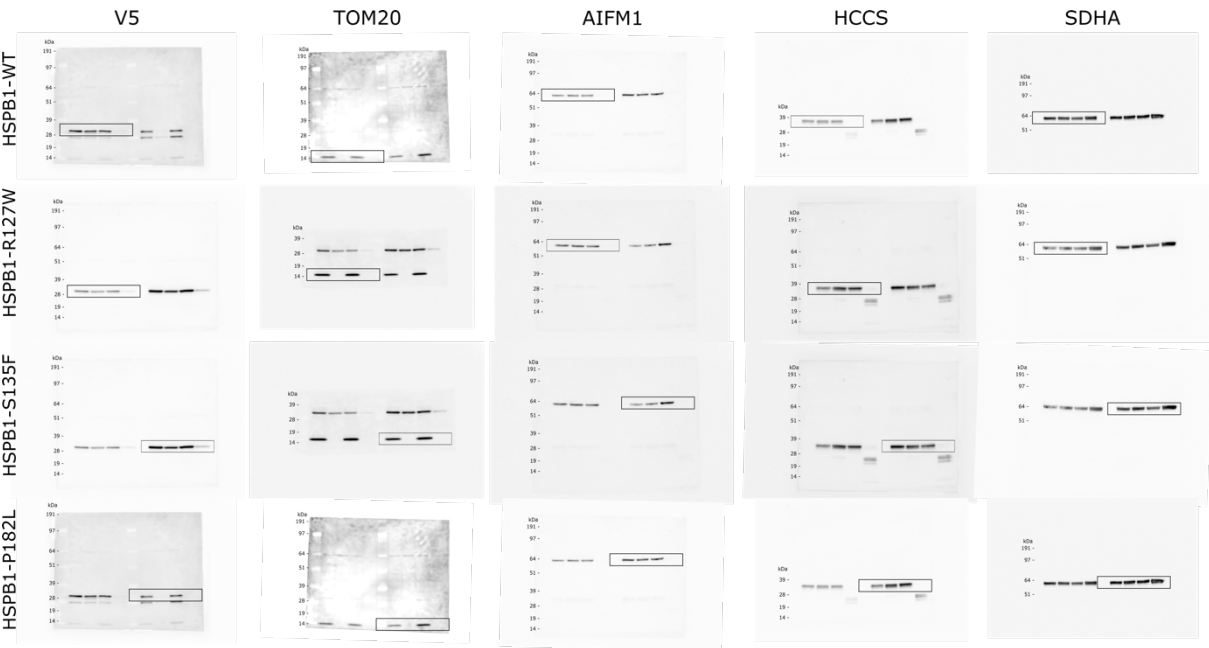

**Figure 6c**

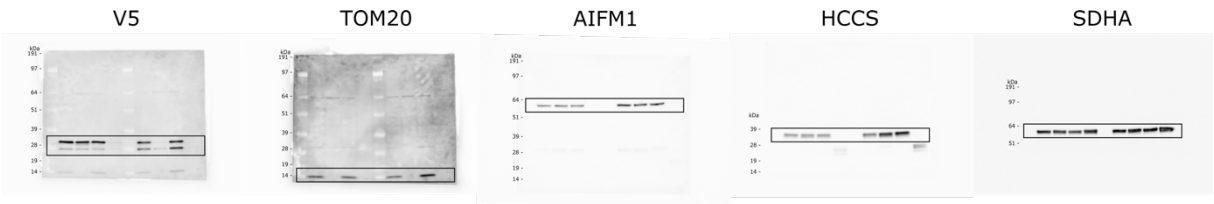

**Figure 6e**

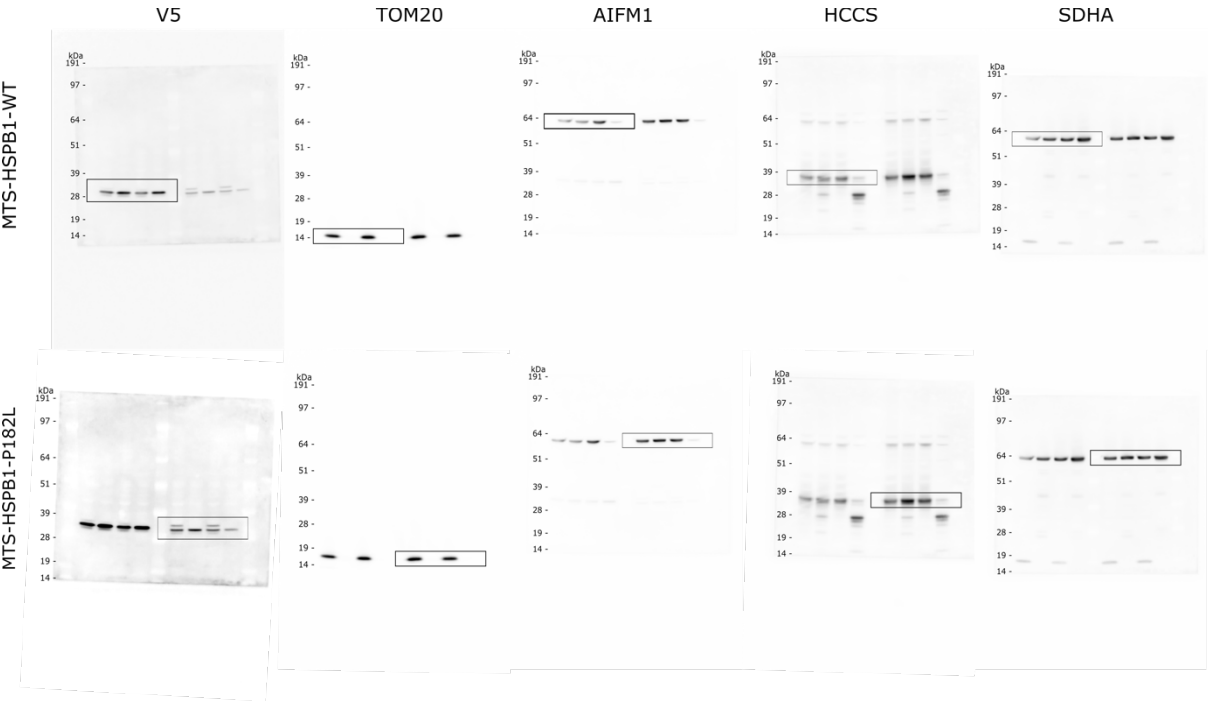

72 **Figure 6f**

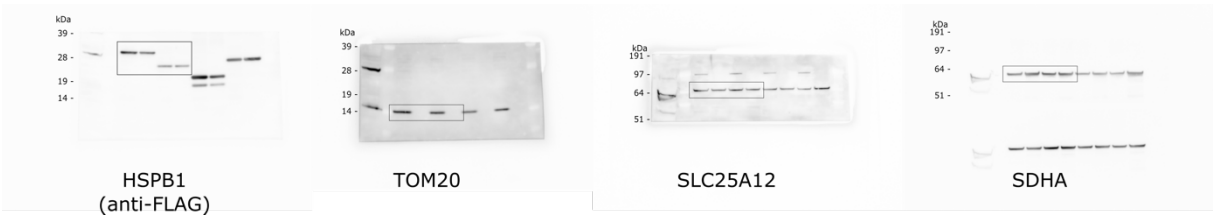

73

74

75

Supplement: Source Data Fig. 6 — Unprocessed western blots. [file 41556_2022_1074_MOESM12_ESM.pdf]

## Extended data Figures

### Extended Data Figure 2a

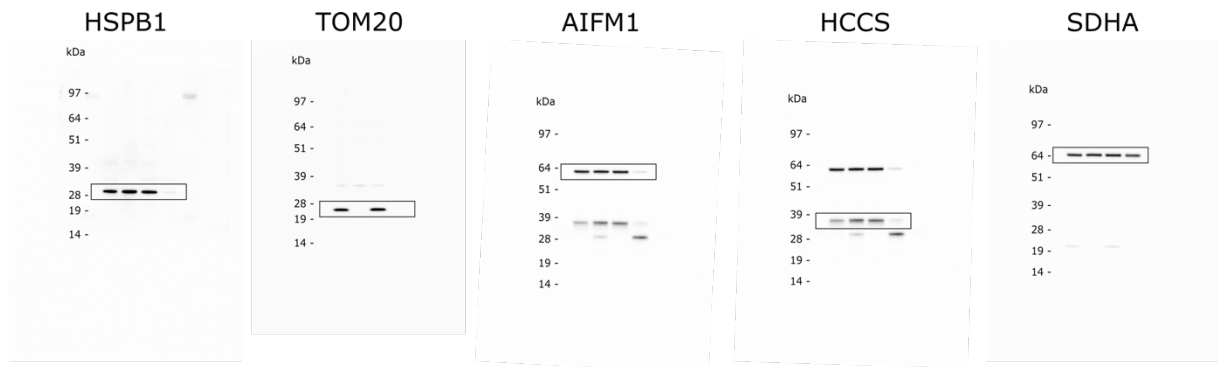

### Extended Data Figure 2b

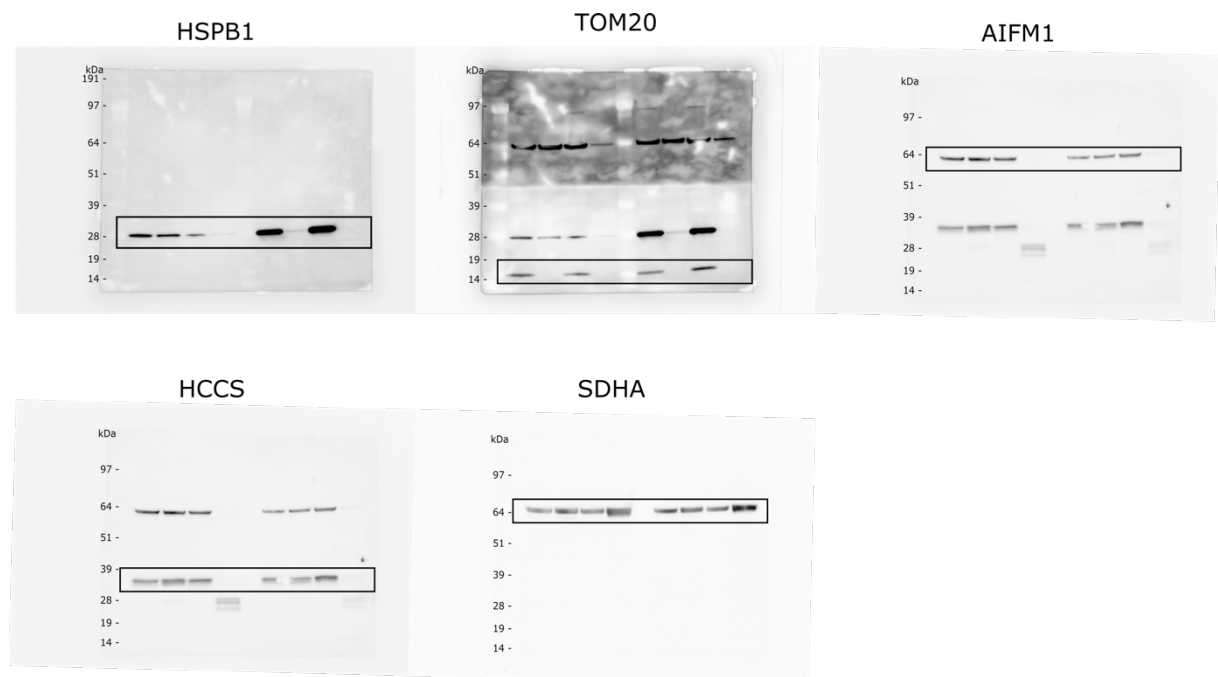

Supplement: Source Data Extended Data Fig./Table 2 — Unprocessed western blots. [file 41556_2022_1074_MOESM15_ESM.pdf]

1

## Extended data Figures

### 2 Extended Data Figure 5a

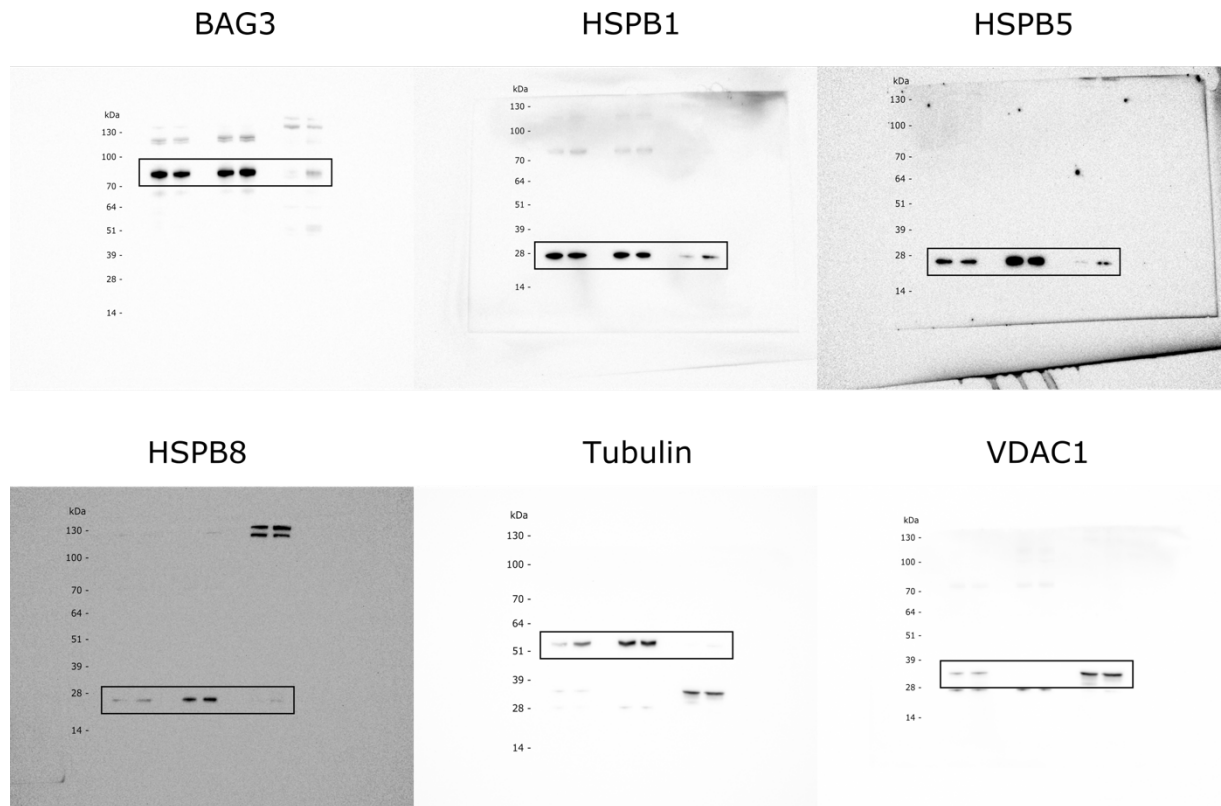

3

4

5

6

7

8

9

10

11

12

13

14

15

16      **Extended Data Figure 5b**

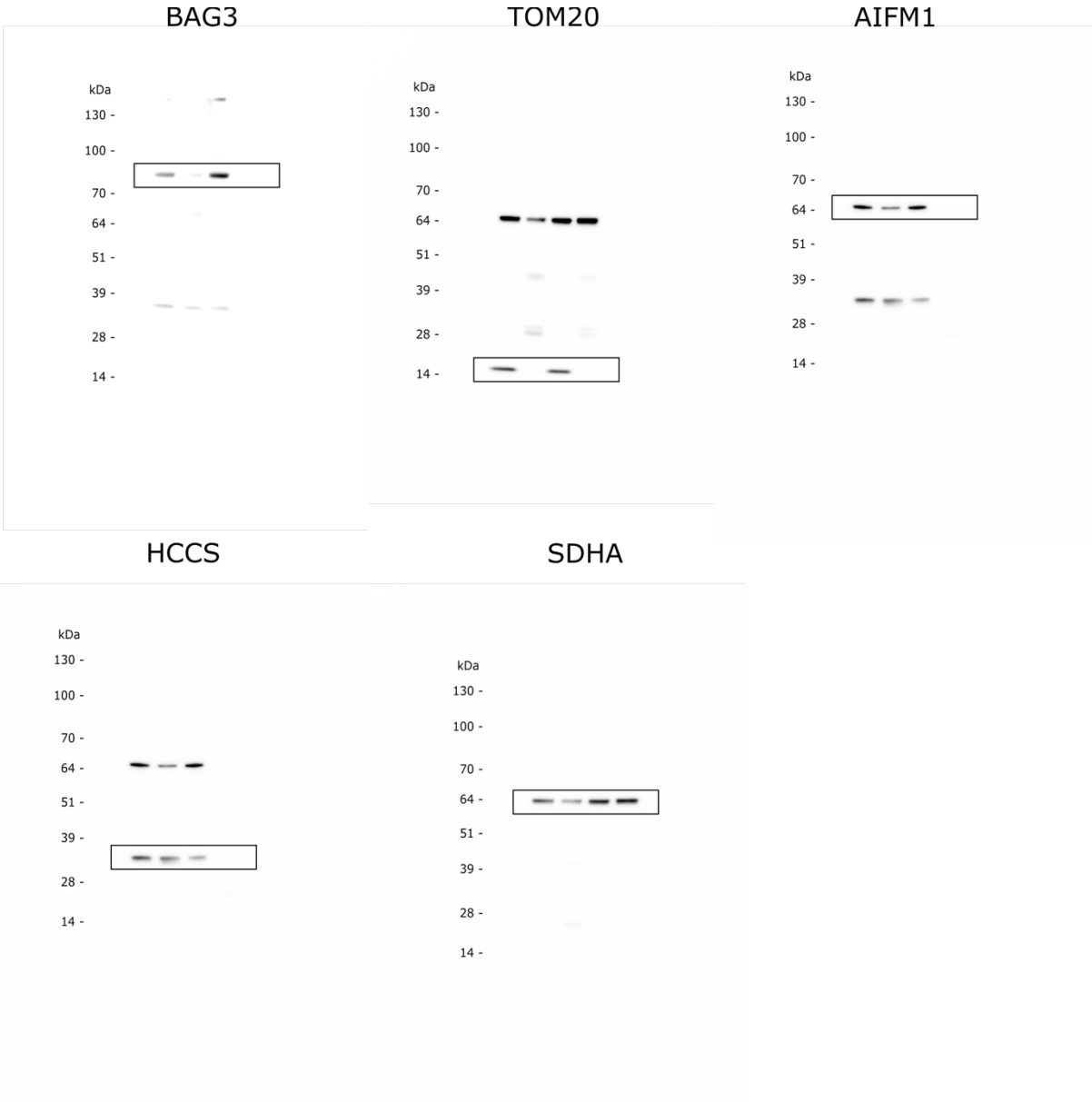

17

18

19

20

21

22

23

24

25

26      **Extended Data Figure 5c**

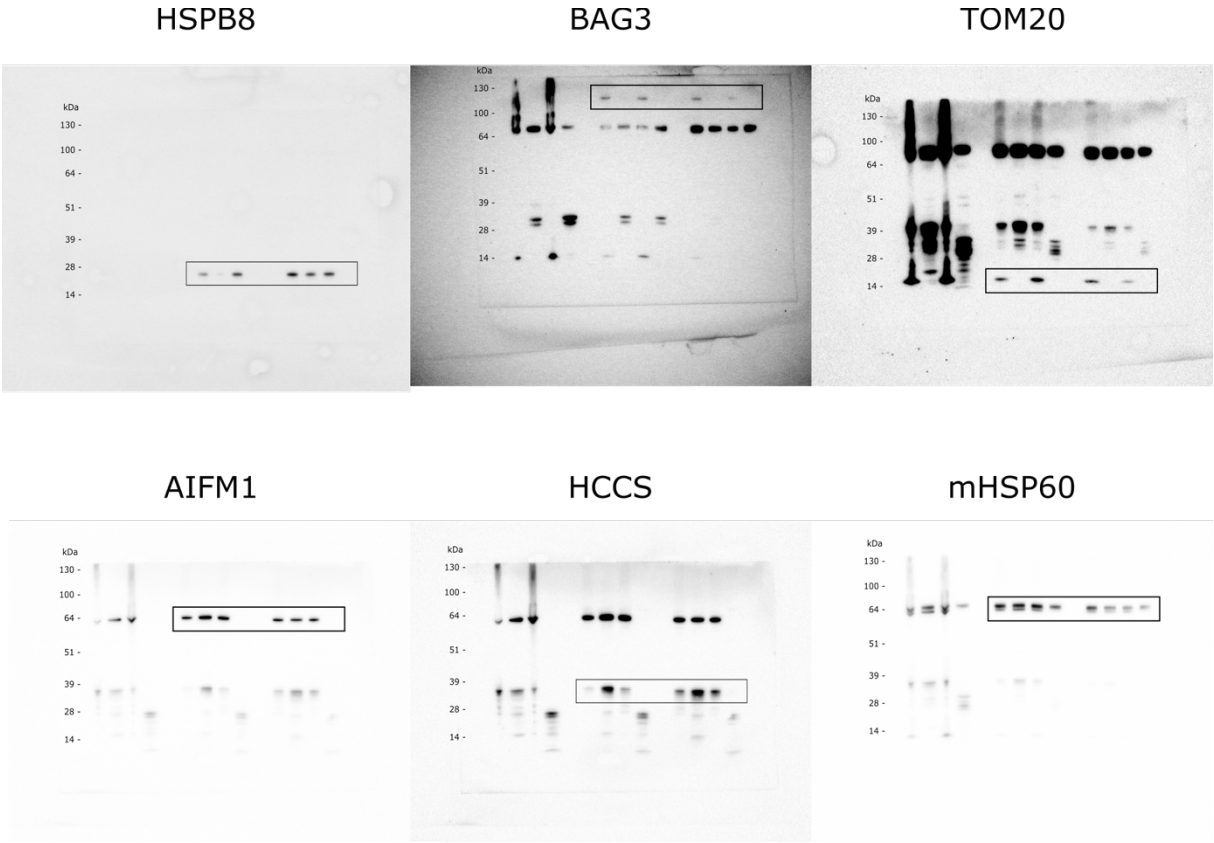

27

28

29

30

31

32

33

34

35

36

37

38

Supplement: Source Data Extended Data Fig./Table 5 — Unprocessed western blots. [file 41556_2022_1074_MOESM18_ESM.pdf]

1

## Extended data Figures

## 2 Extended Data Figure 7a

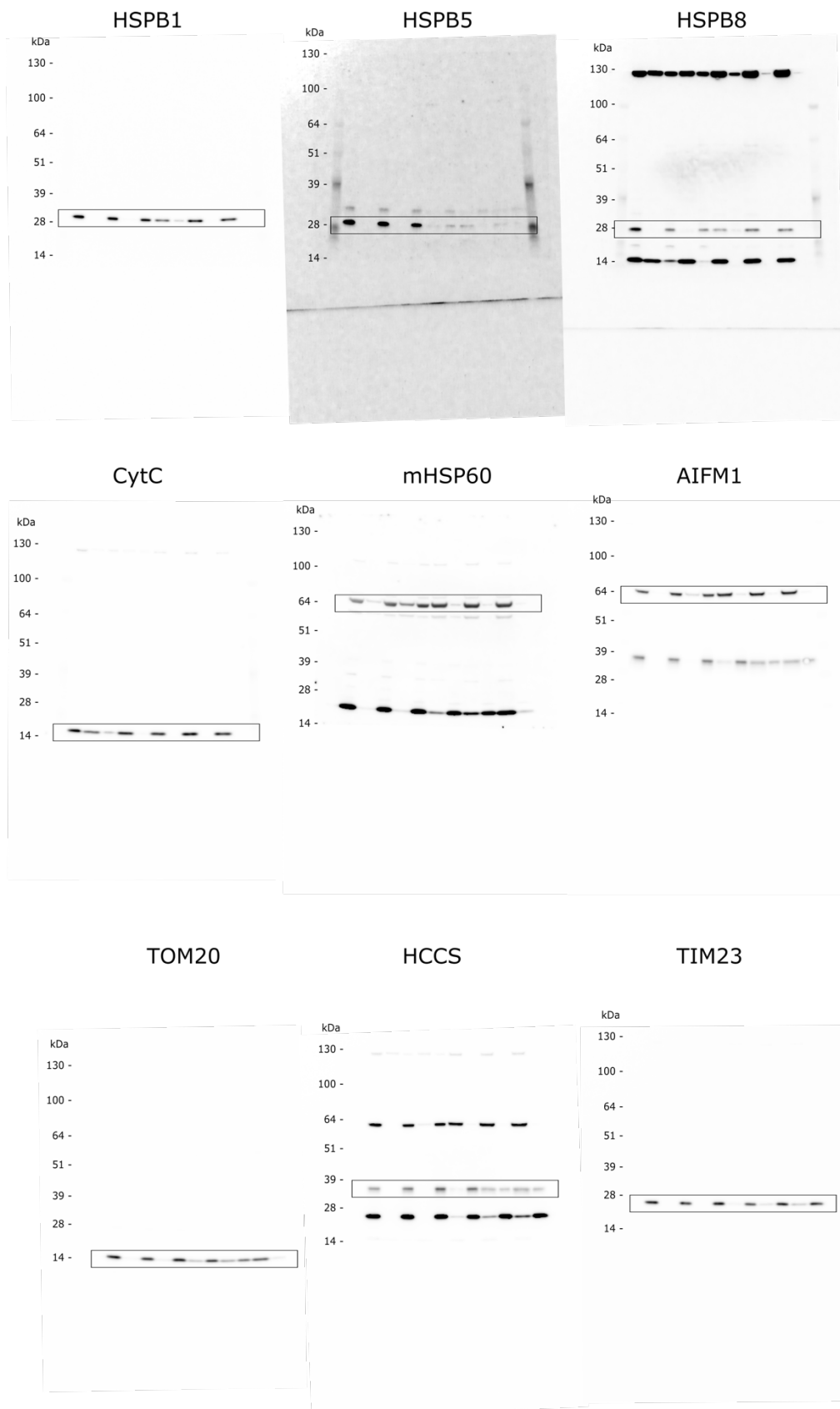

3

Supplement: Source Data Extended Data Fig./Table 7 — Unprocessed western blots. [file 41556_2022_1074_MOESM19_ESM.pdf]

1

Extended data Figures

2 Extended Data Figure 10a

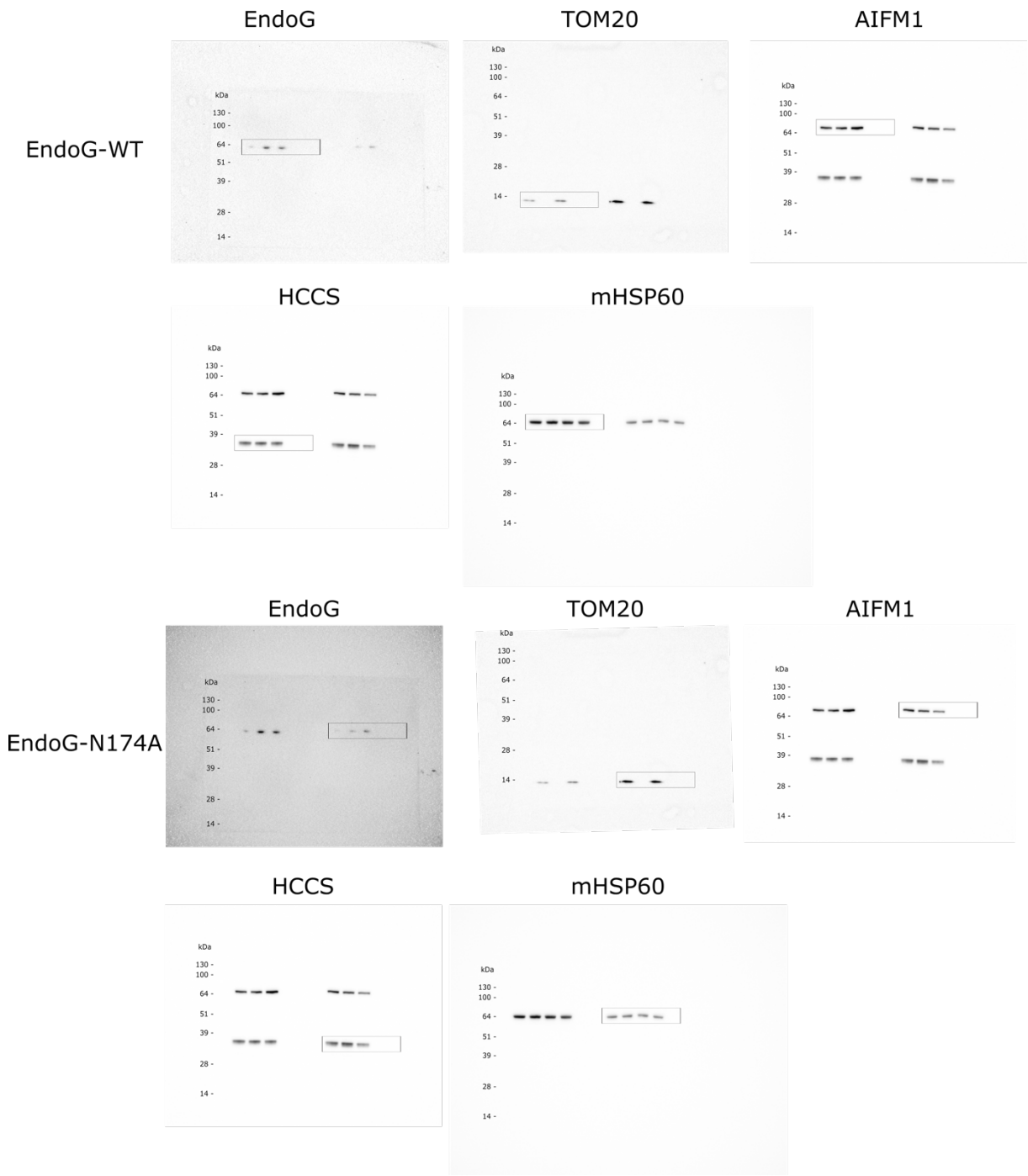

3

4

5

6

7

8    **Extended Data Figure 10b**

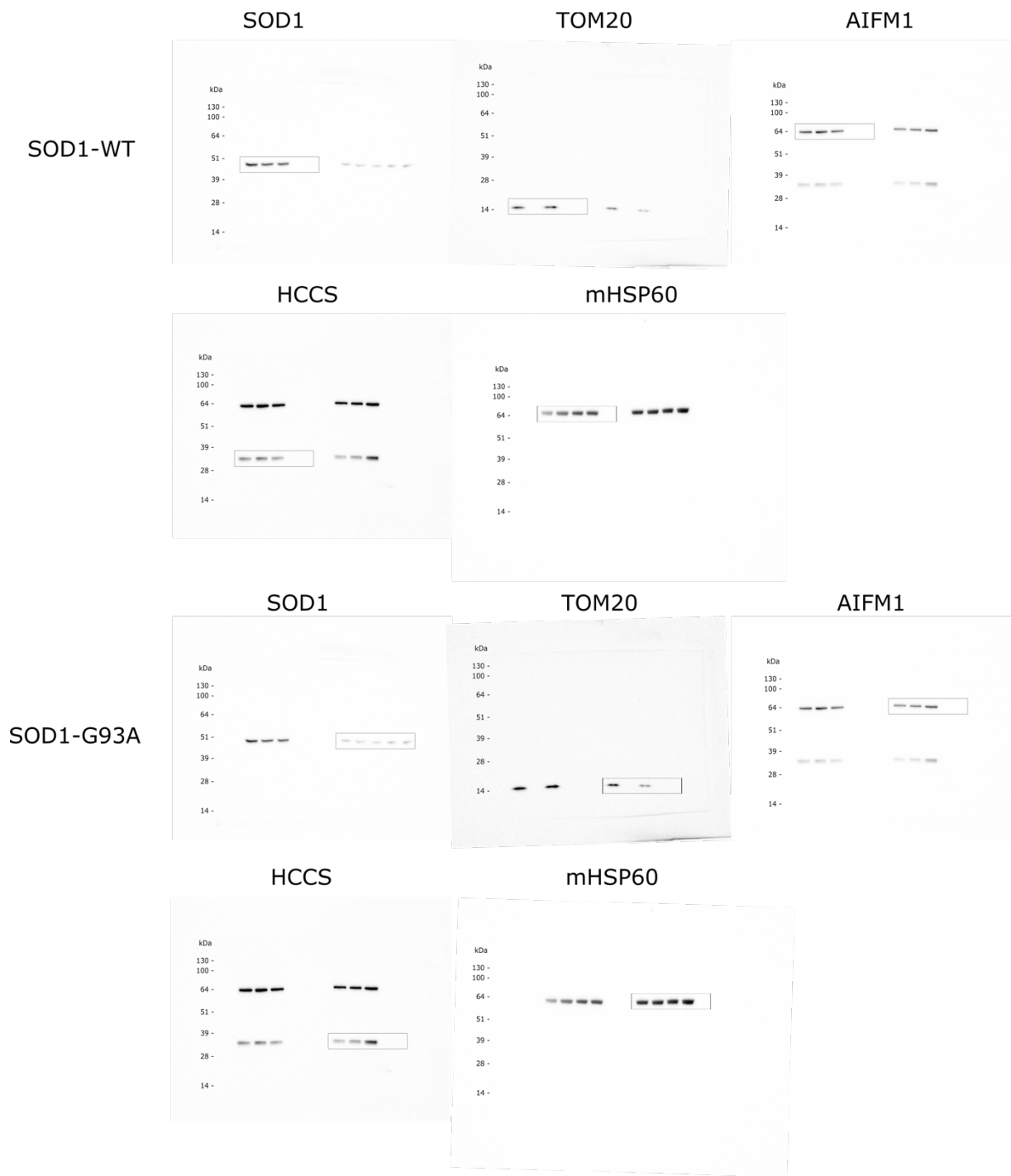

Supplement: Source Data Extended Data Fig./Table 10 — Unprocessed western blots. [file 41556_2022_1074_MOESM20_ESM.pdf]
