# Supplementary material for: Small heat shock proteins operate as molecular chaperones in the mitochondrial intermembrane space
Source: Nat Cell Biol. 2023 Jan 23;25(3):467–80. doi: 10.1038/s41556-022-01074-9 (PMC10014586; doi:10.1038/s41556-022-01074-9)

Source data files (Extended figures)

Extended Data Figure 1a

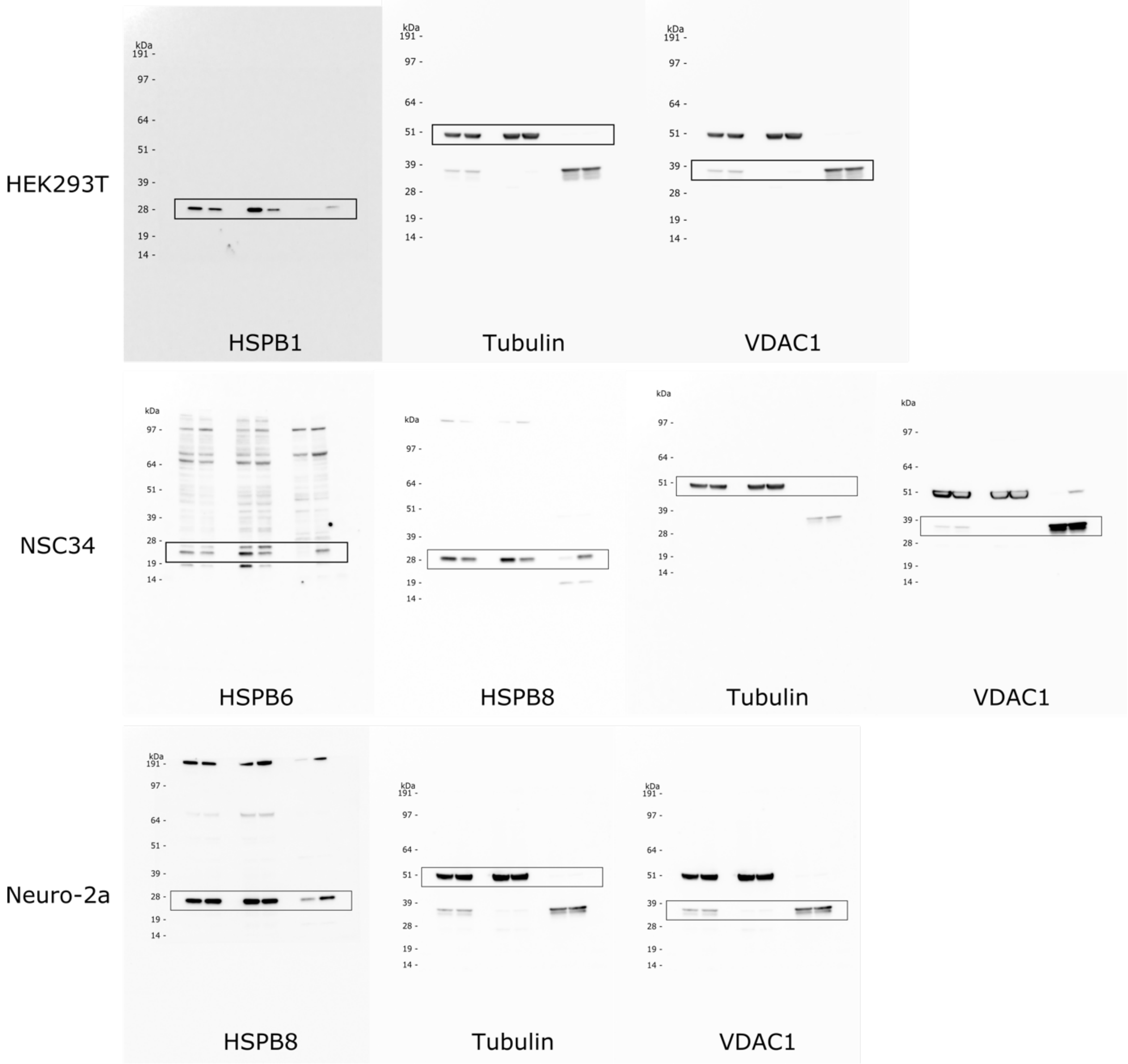

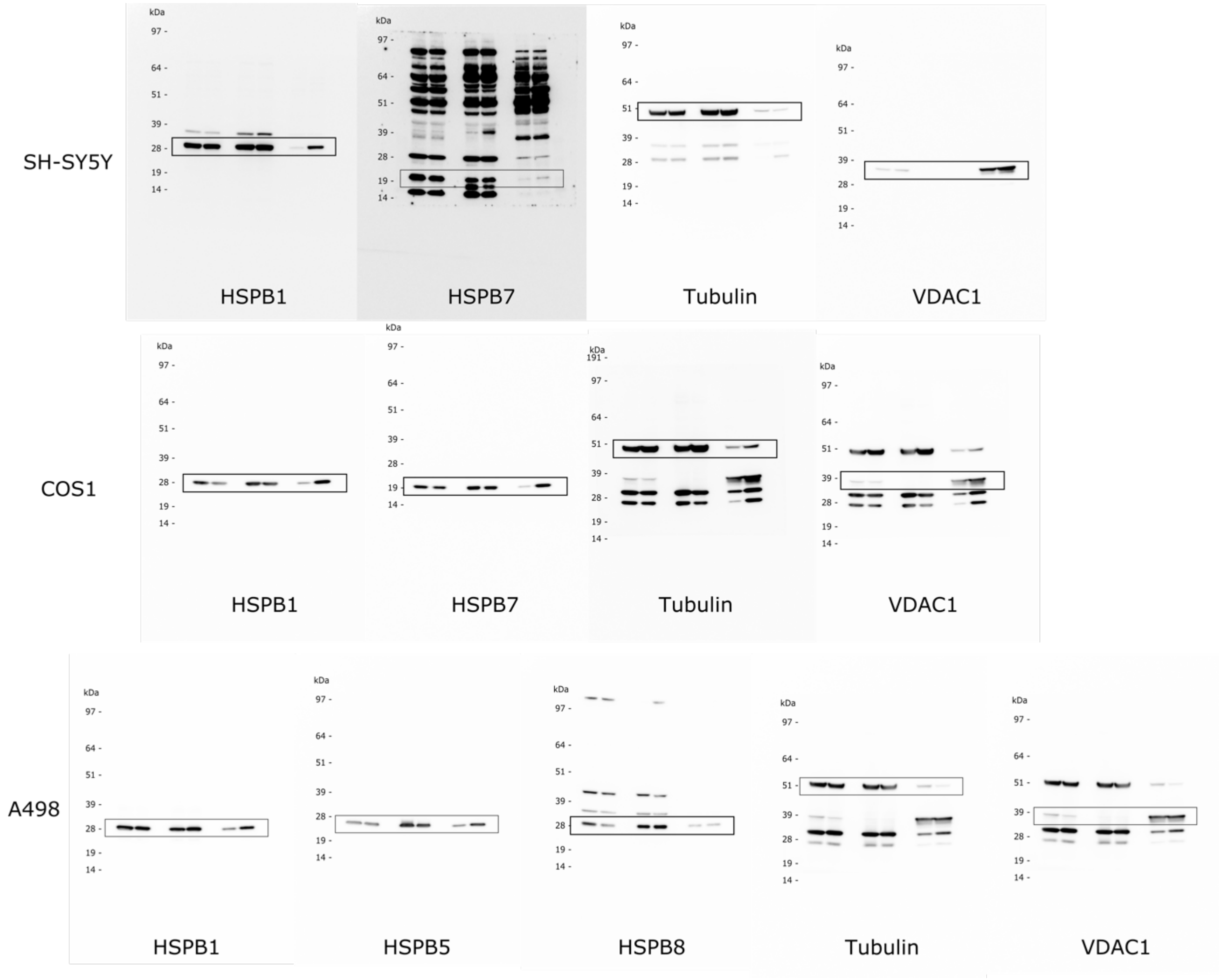
SH-SY5Y
HSPB1
HSPB7
Tubulin
VDAC1
COS1
HSPB1
HSPB7
Tubulin
VDAC1
A498
HSPB1
HSPB5
HSPB8
Tubulin
VDAC1

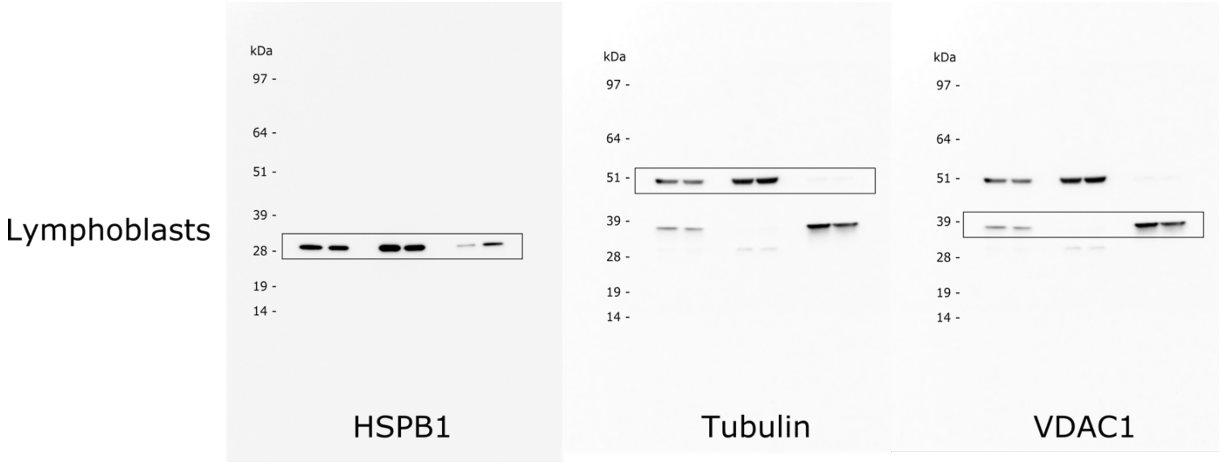
Lymphoblasts
kDa
97 -
64 -
51 -
39 -
28 -
19 -
14 -
HSPB1
Tubulin
VDAC1

Extended Data Figure 1b

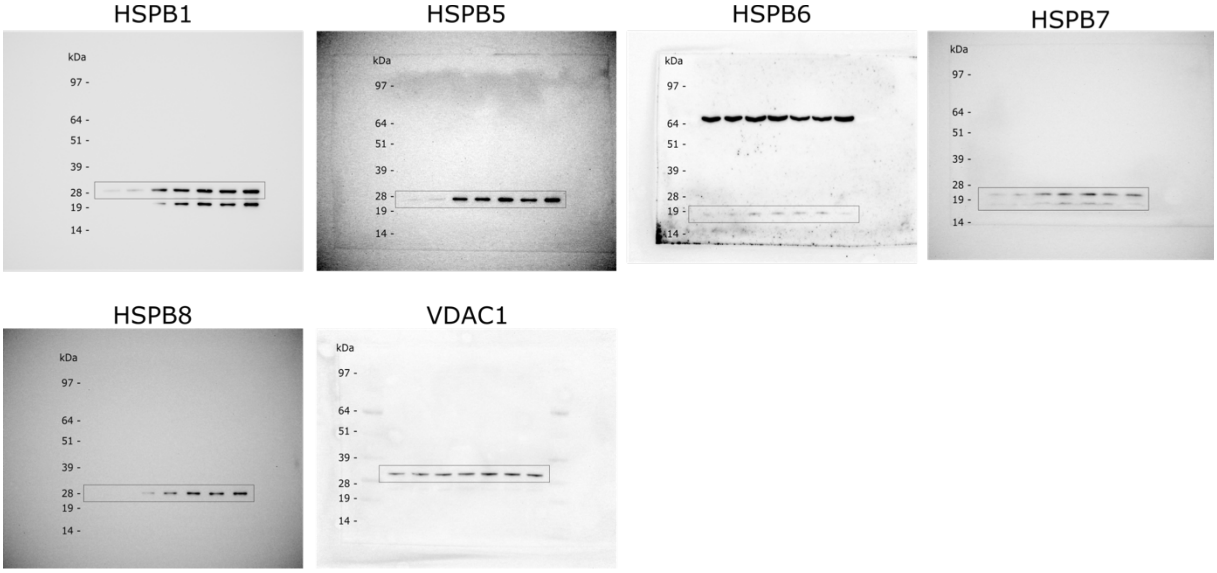

Supplement: Source Data Extended Data Fig./Table 1 — Unprocessed western blots. [file 41556_2022_1074_MOESM14_ESM.pdf]
