# Supplementary material for: Small heat shock proteins operate as molecular chaperones in the mitochondrial intermembrane space
Source: Nat Cell Biol. 2023 Jan 23;25(3):467–80. doi: 10.1038/s41556-022-01074-9 (PMC10014586; doi:10.1038/s41556-022-01074-9)

Extended data Figures

Extended Data Figure 3

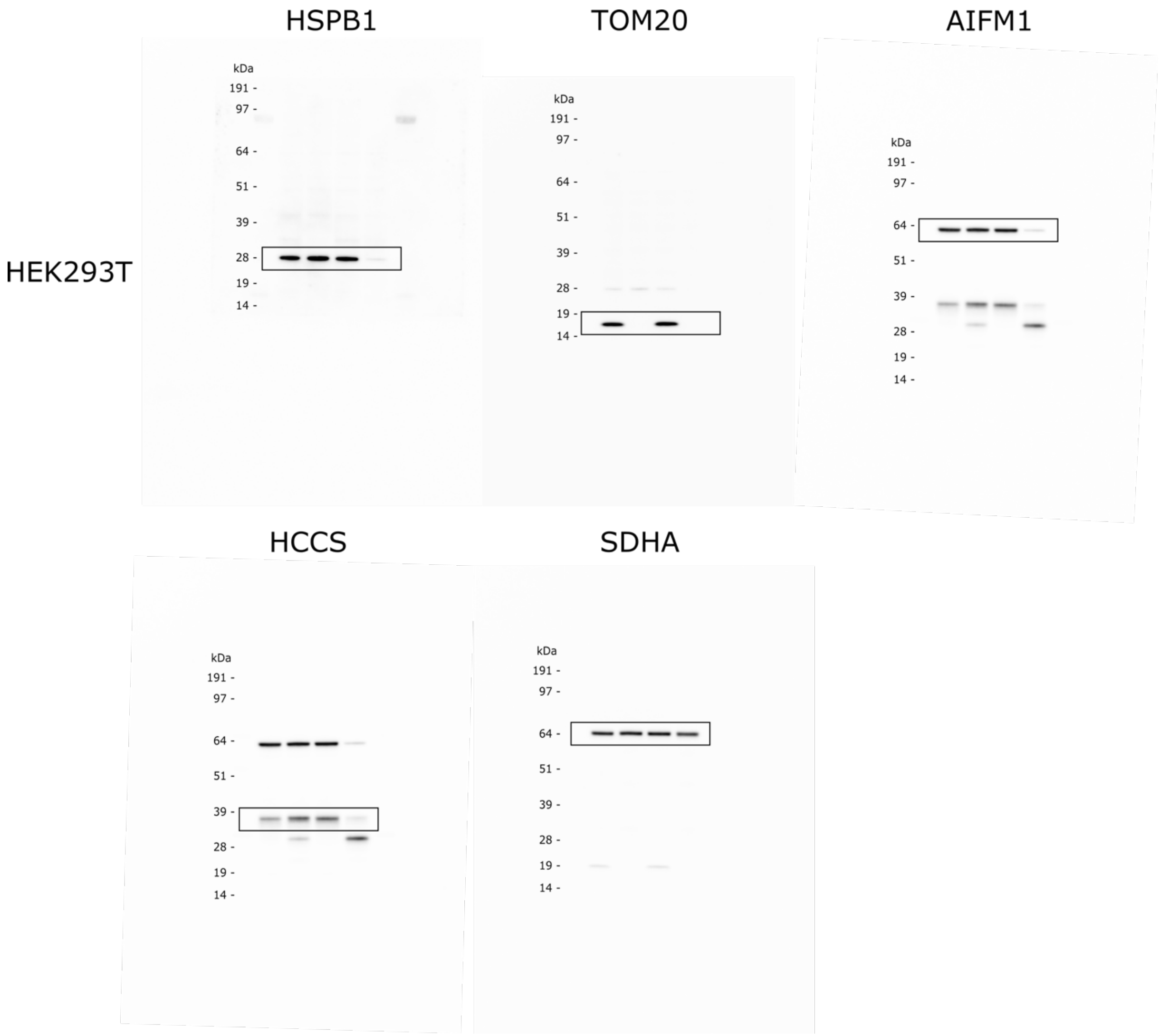

Extended Data Figure 3 (continued)

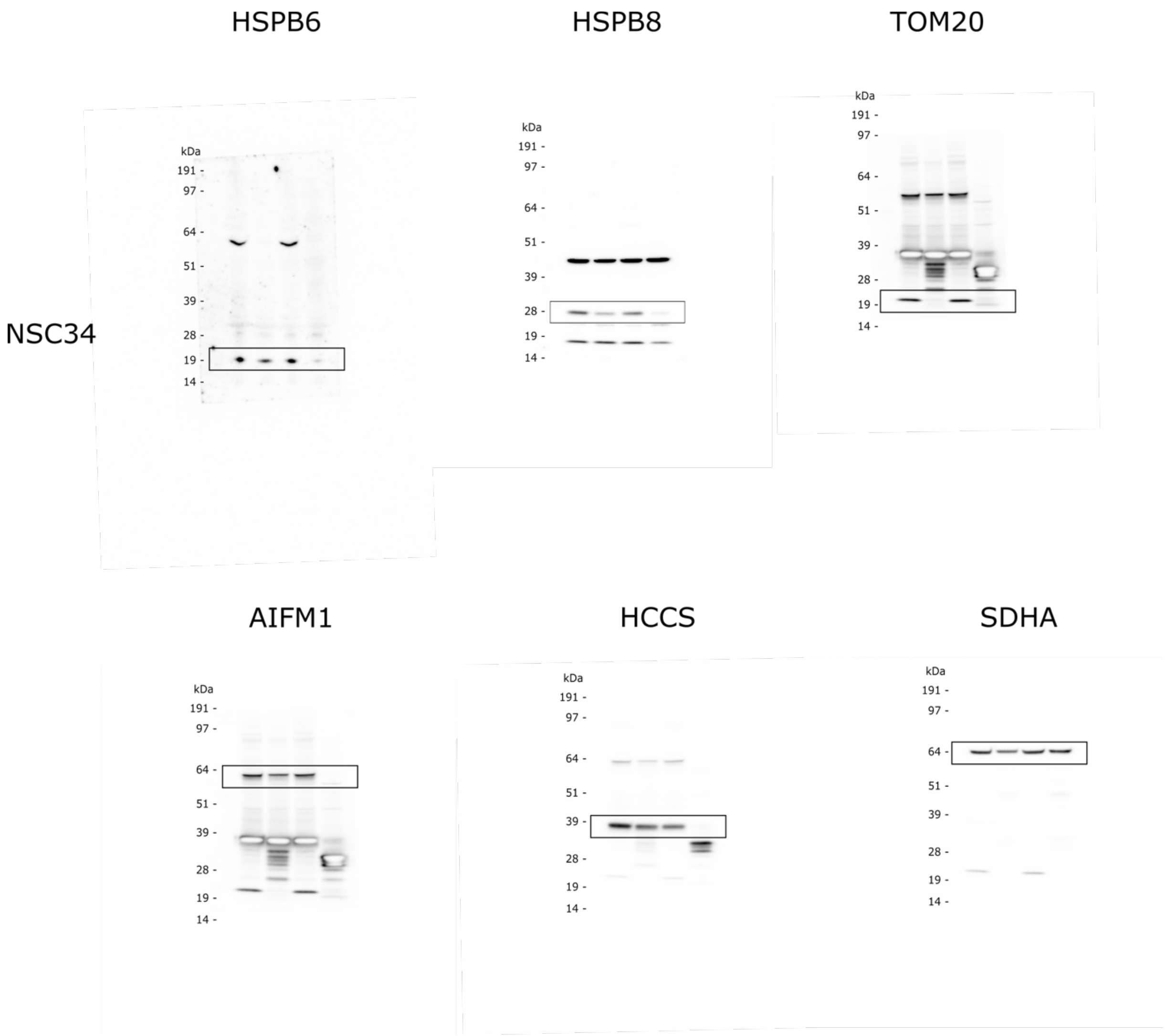

Extended Data Figure 3 (continued)

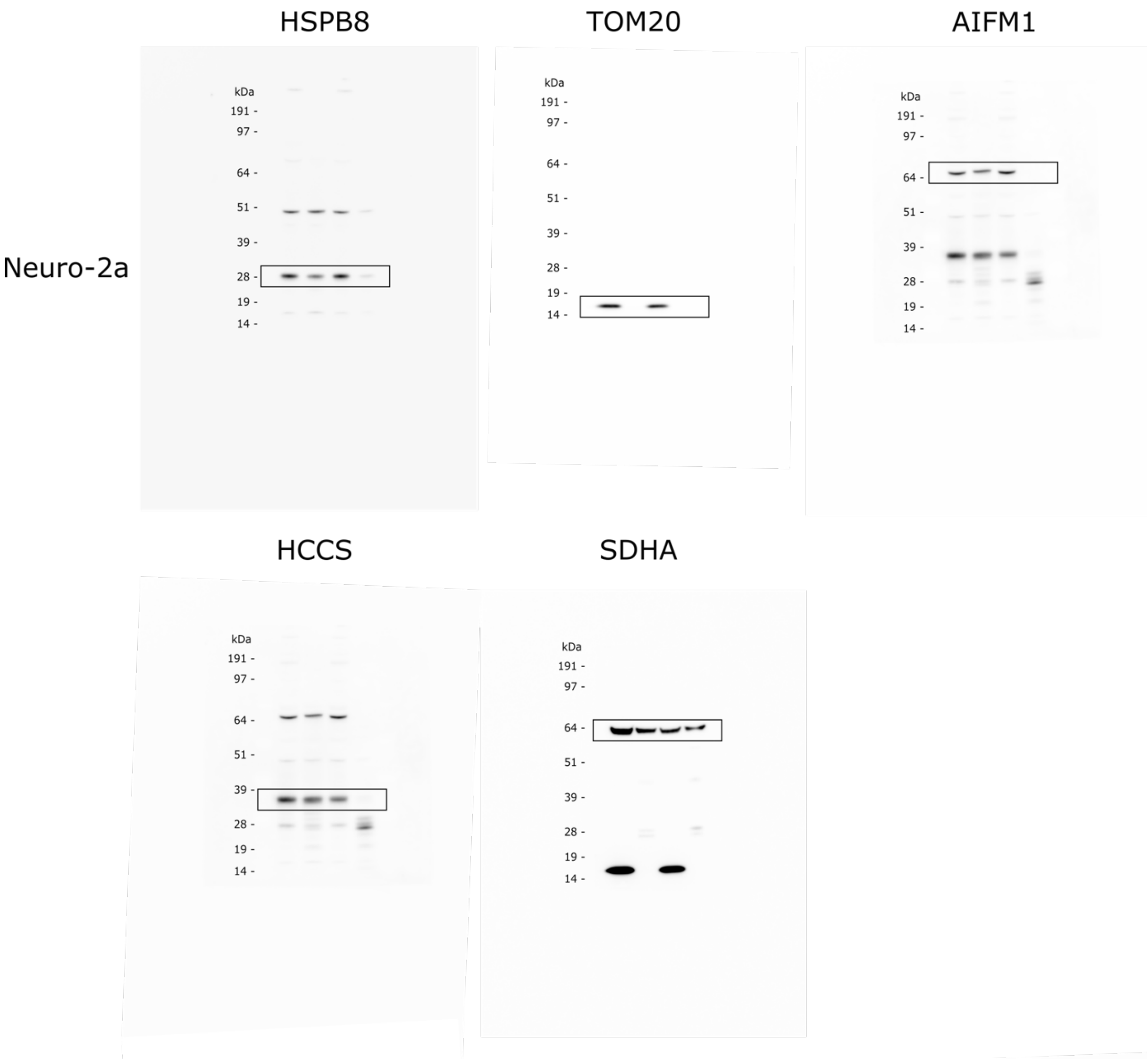

Extended Data Figure 3 (continued)

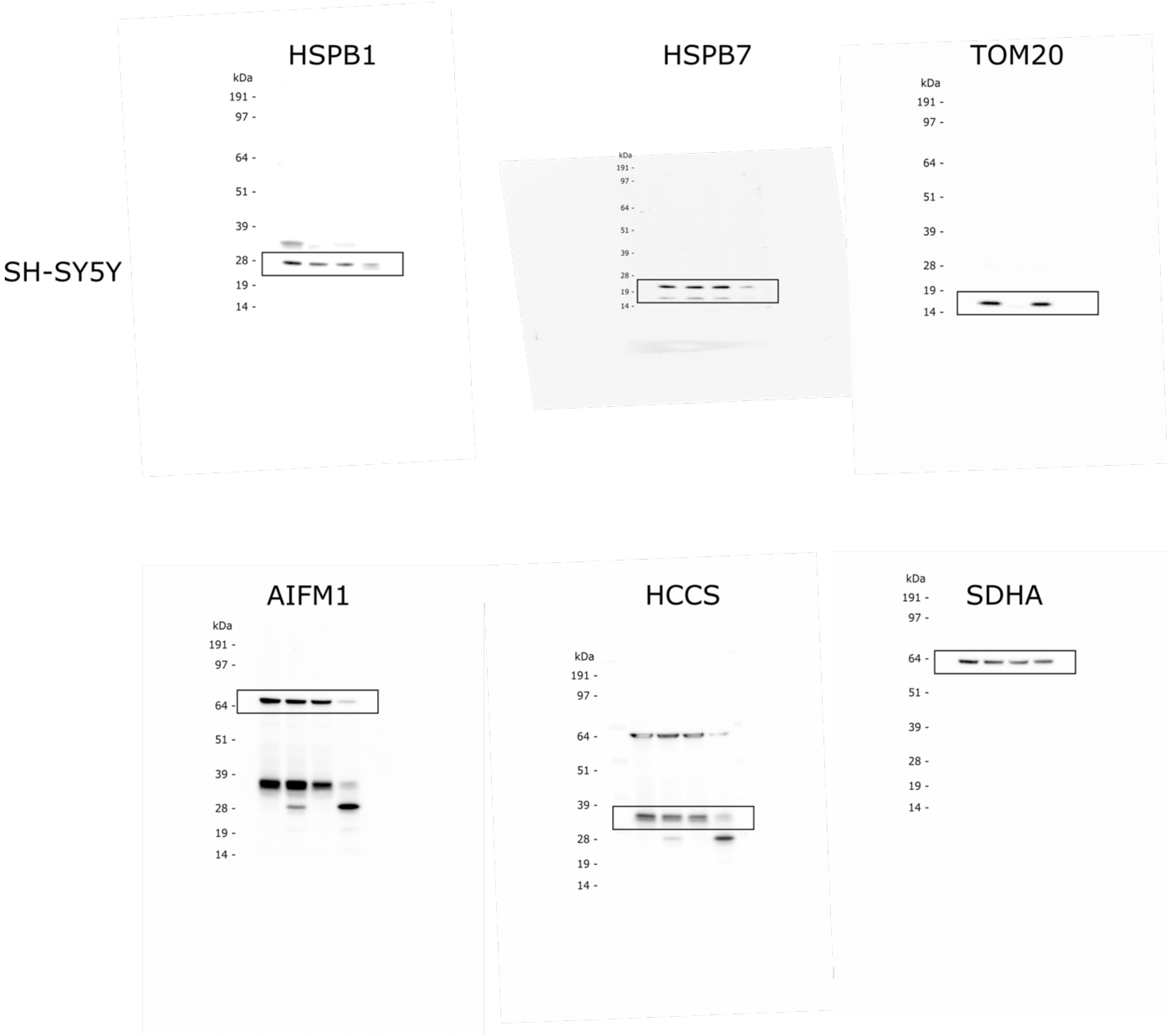

Extended Data Figure 3 (continued)

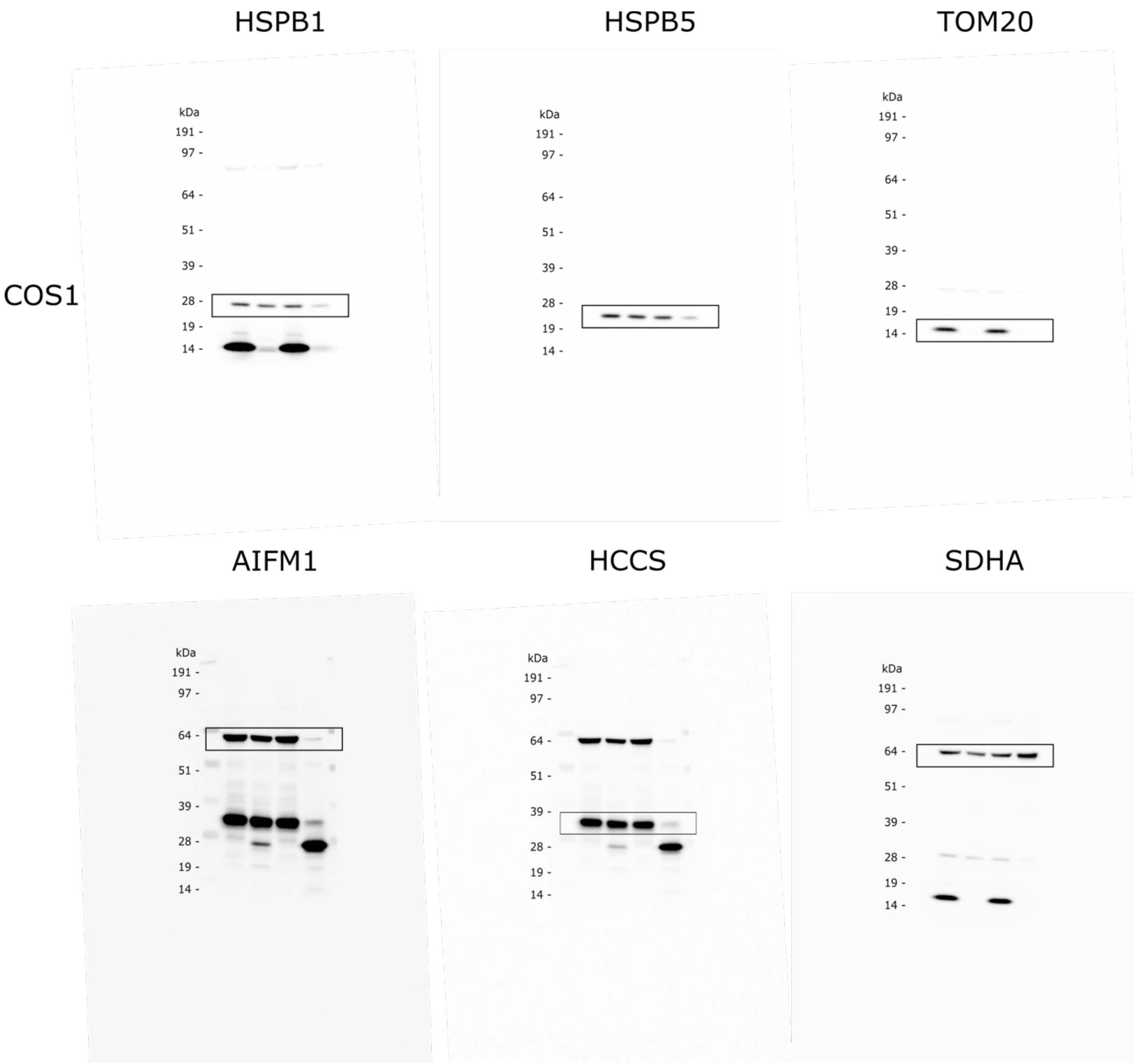

Extended Data Figure 3 (continued)

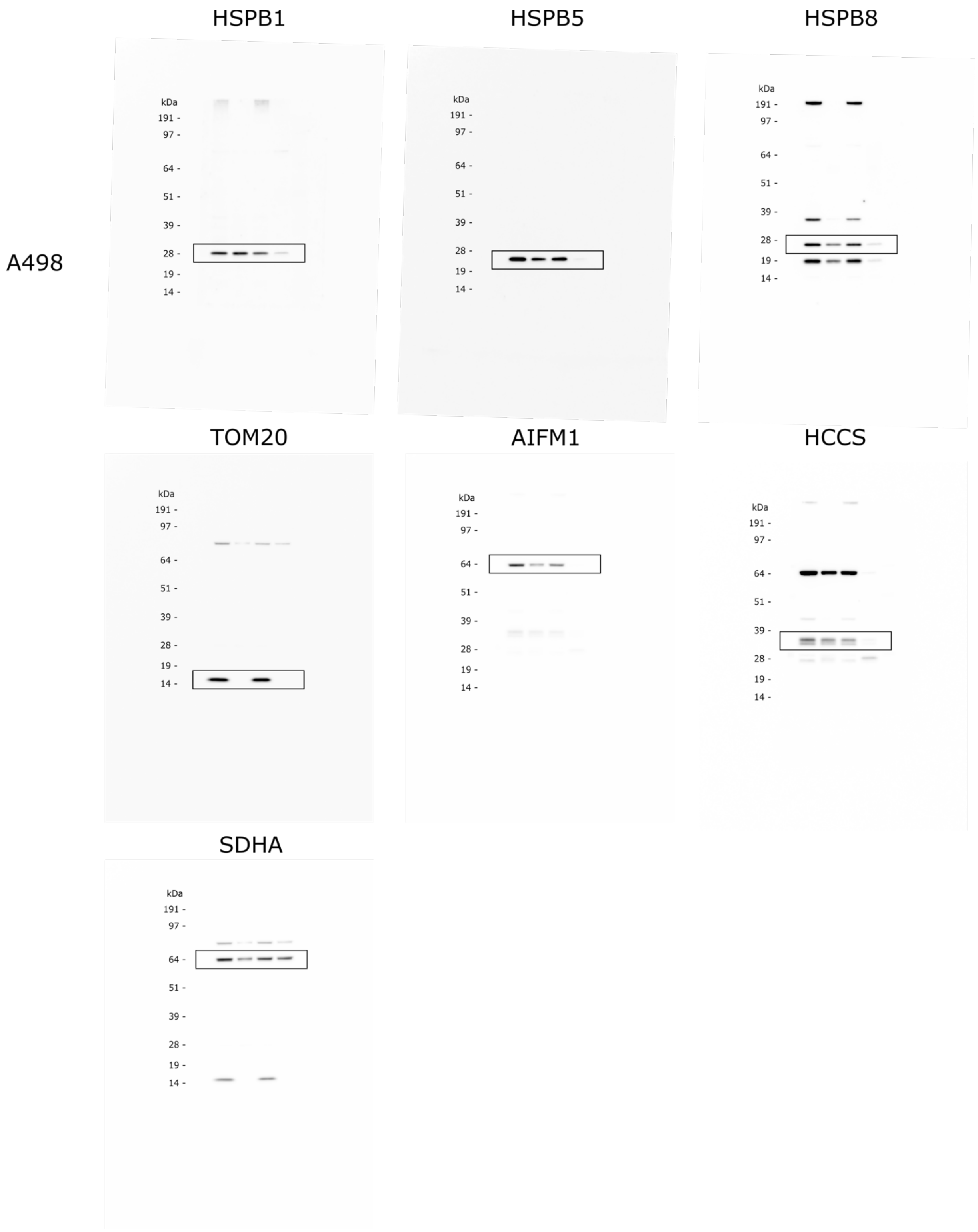

Extended Data Figure 3 (continued)

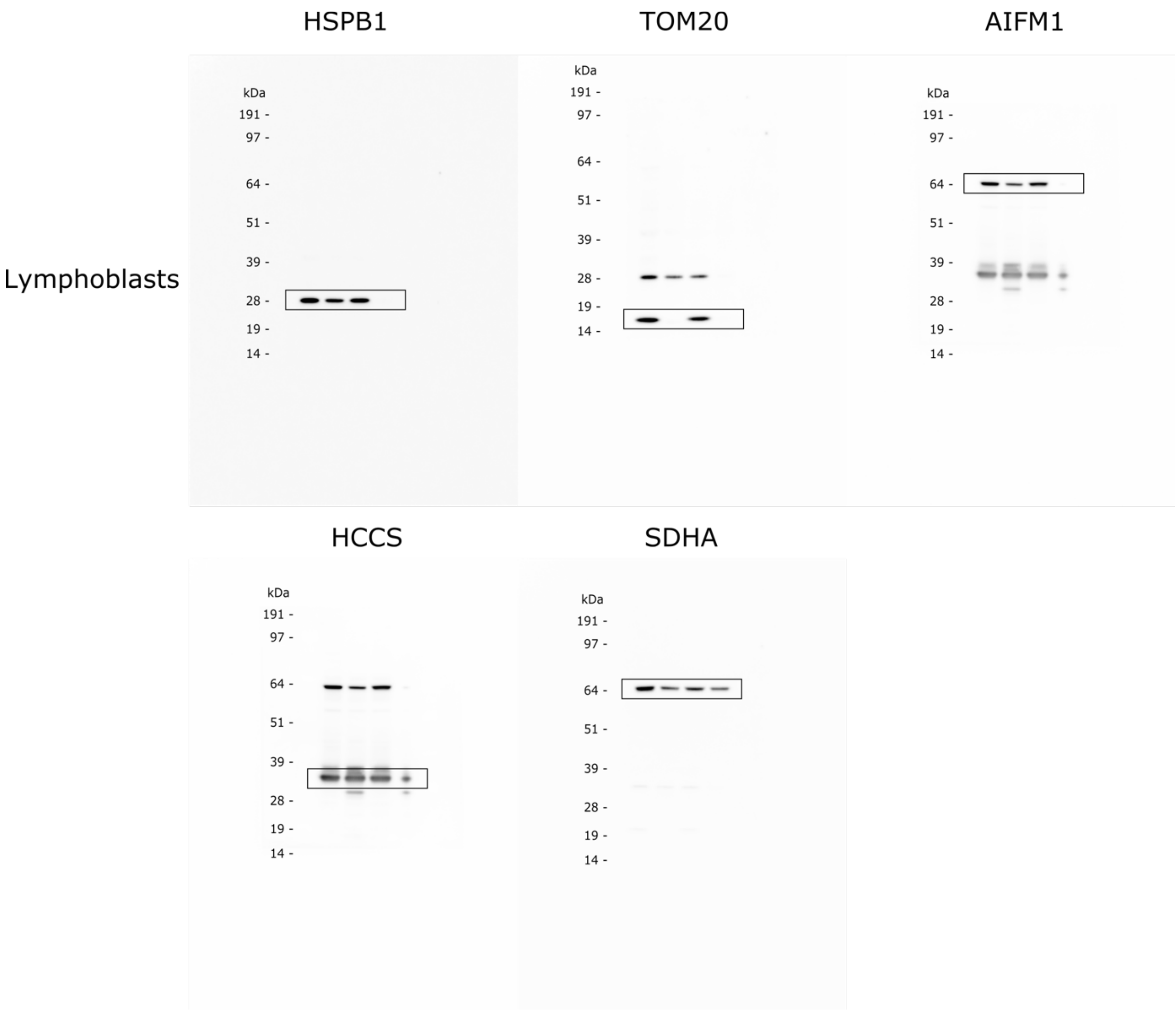

Extended Data Figure 3 (continued)

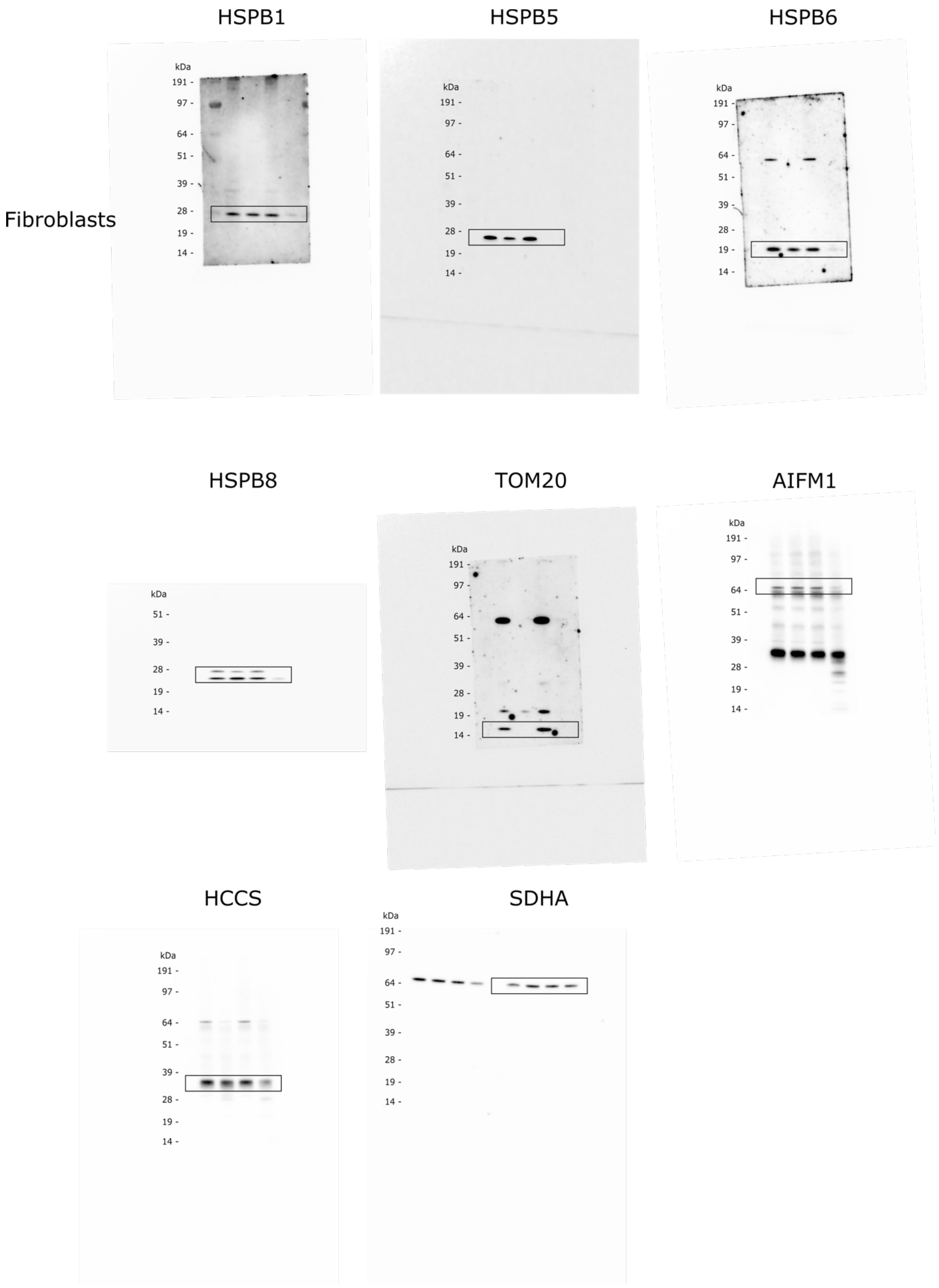

Extended Data Figure 3 (continued)

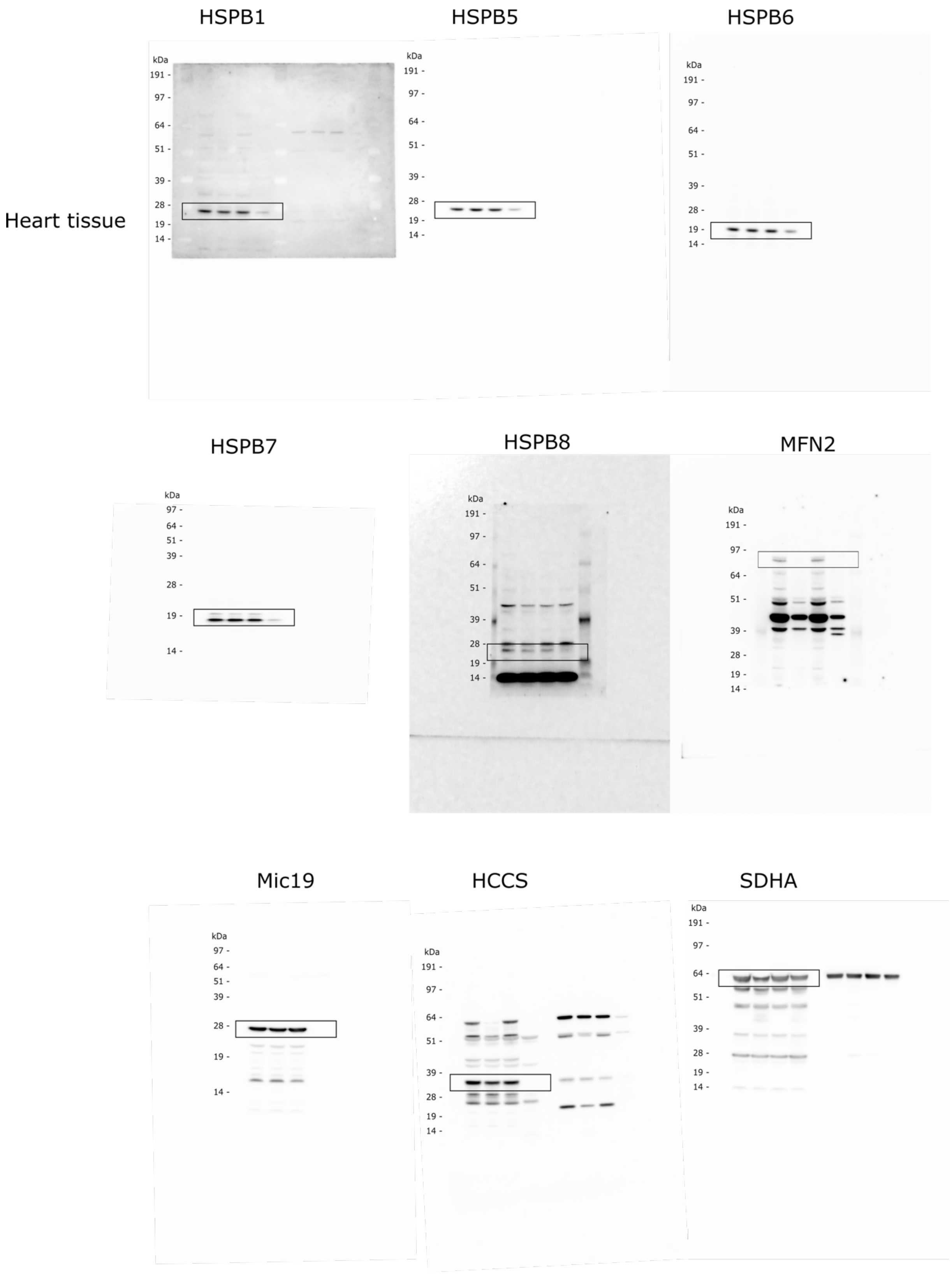

Supplement: Source Data Extended Data Fig./Table 3 — Unprocessed western blots. [file 41556_2022_1074_MOESM16_ESM.pdf]
